# Supplementary figures and images for: Cohesin reconstitution and homologous recombination repair of DNA double-strand breaks in late mitosis
Source: eLife. 2025 Nov 11;13:RP92706. doi: 10.7554/eLife.92706 (PMC12604858; doi:10.7554/eLife.92706)

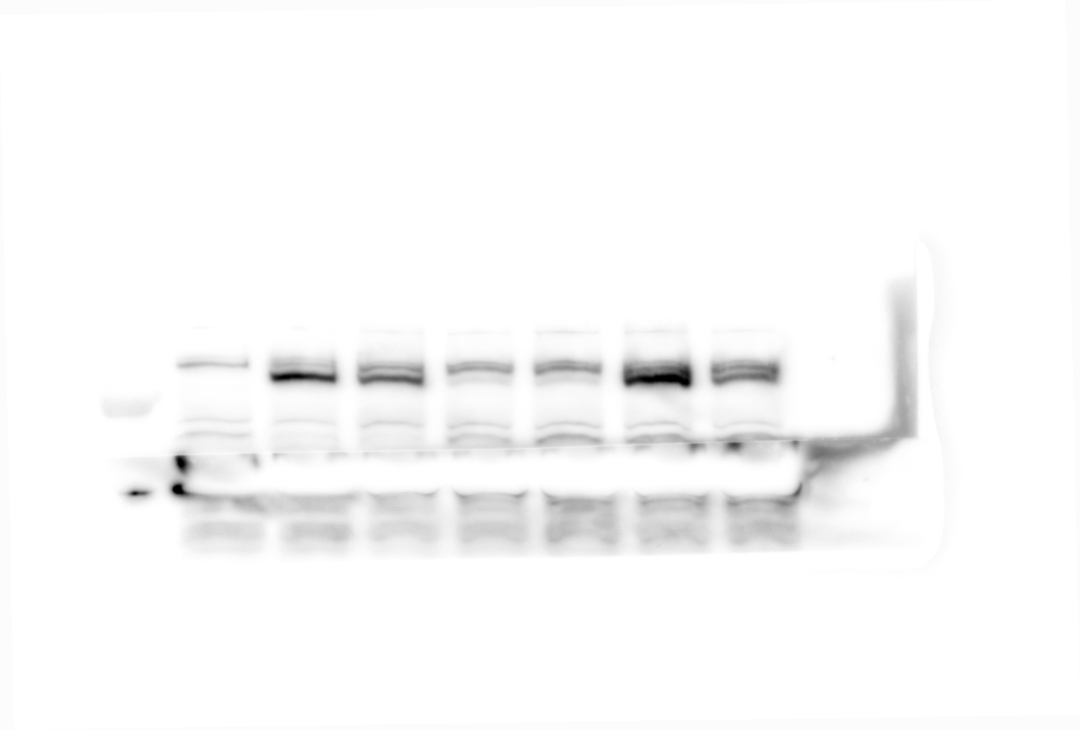

Supplement: Figure 1—source data 1. [file elife-92706-fig1-data1.zip › Figure 1 - source data 1_Original blots/Fig1B_r1_myc.tiff]

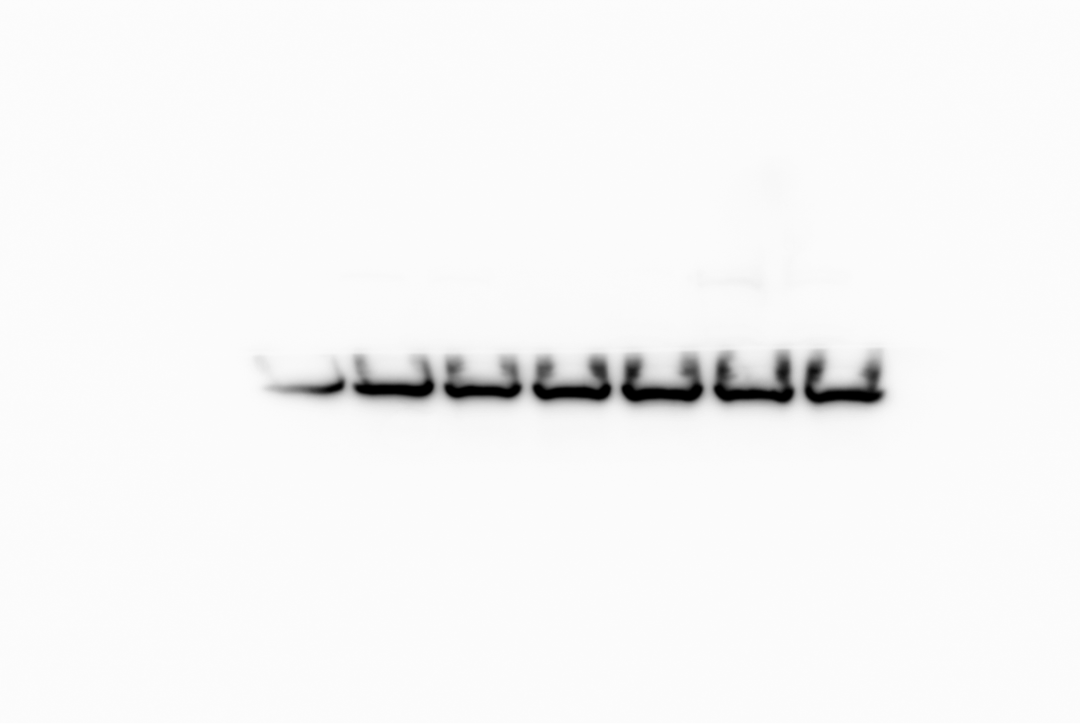

Supplement: Figure 1—source data 1. [file elife-92706-fig1-data1.zip › Figure 1 - source data 1_Original blots/Fig1B_r1_PGK1.tiff]

Figure 1b – Western blots

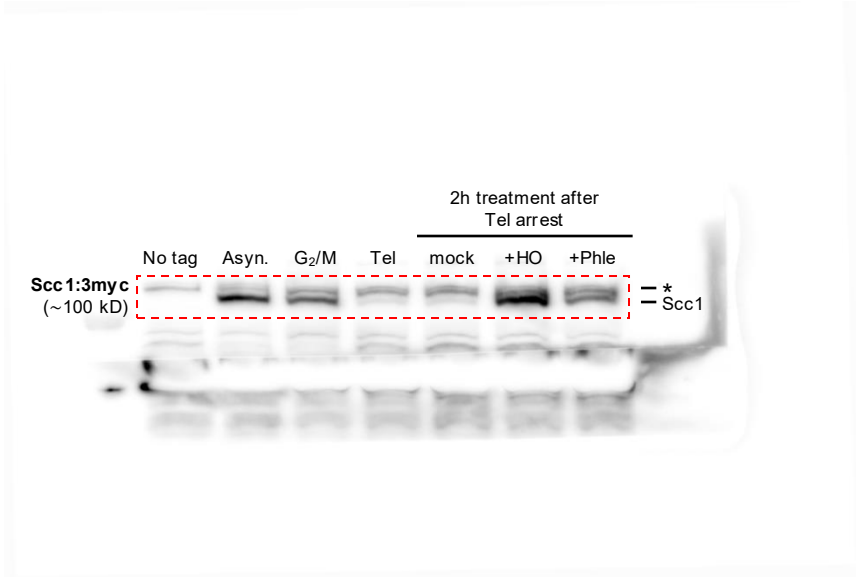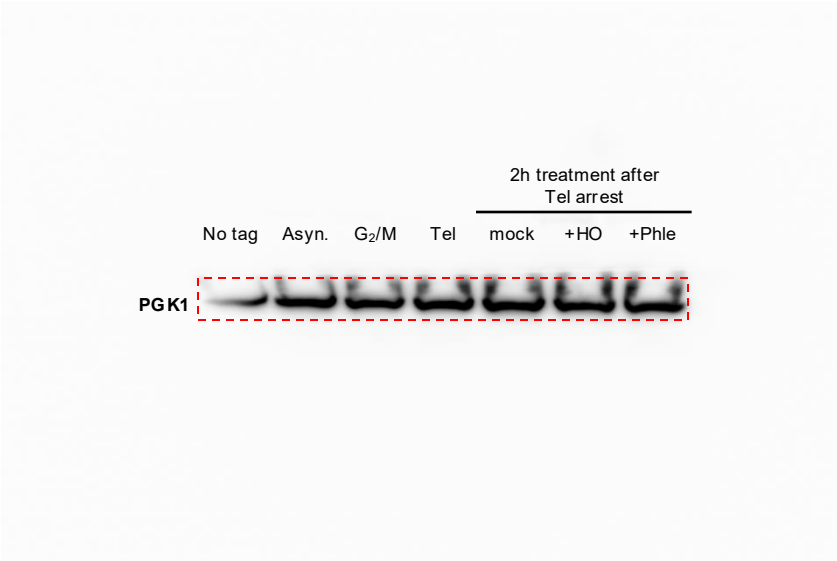

Supplement: Figure 1—source data 2. [file elife-92706-fig1-data2.pdf]

Fig 1C and D

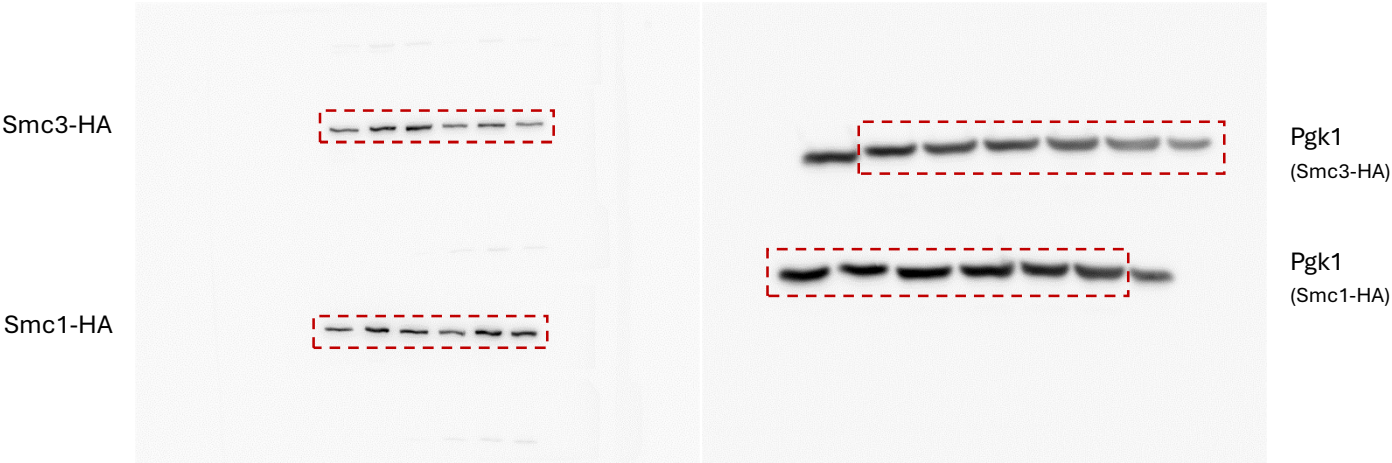

Supplement: Figure 1—source data 3. [file elife-92706-fig1-data3.pdf]

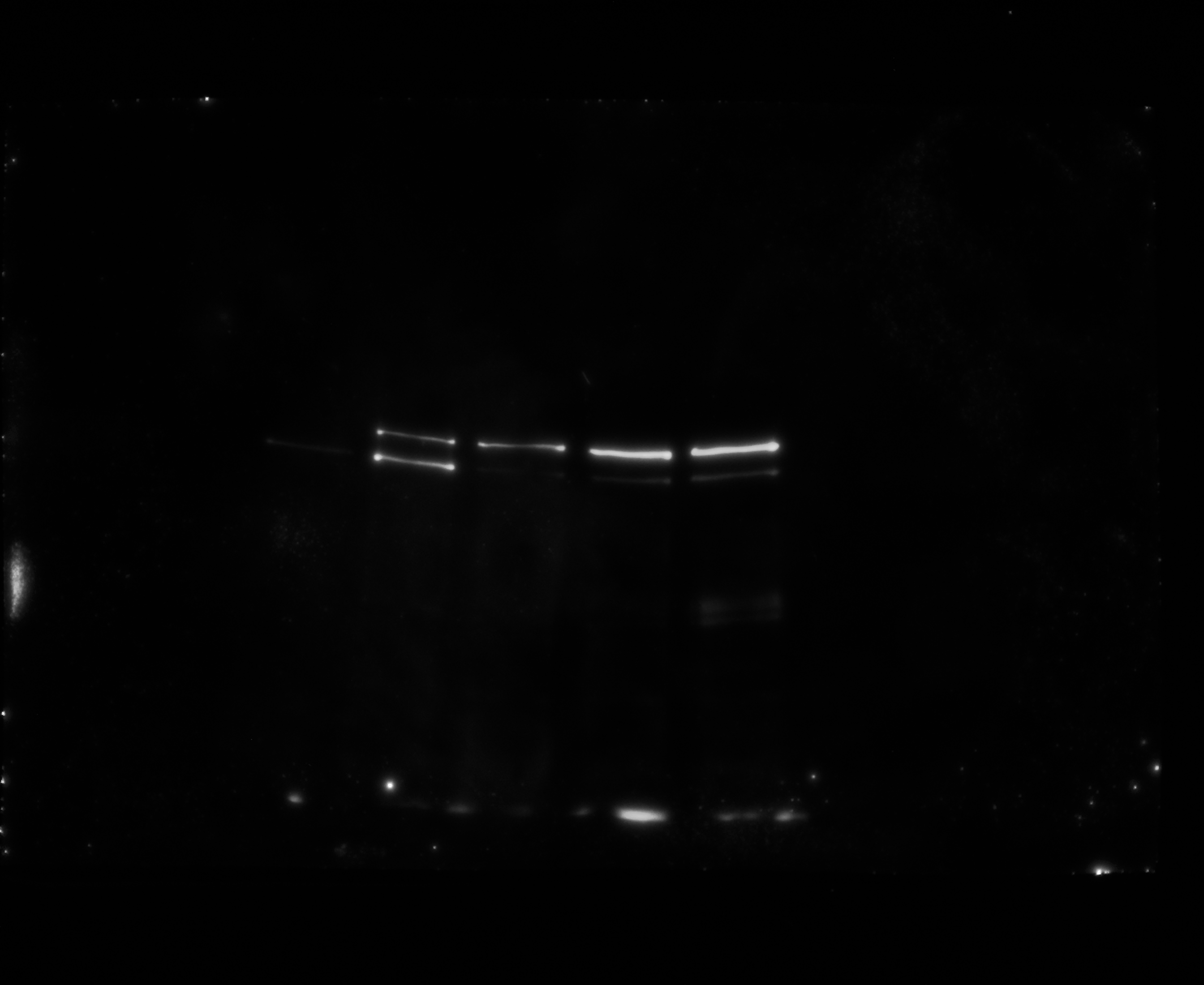

Supplement: Figure 2—source data 1. [file elife-92706-fig2-data1.zip › Figure 2 - source data 1_Original blots/Figure2A acSmc3.Tif]

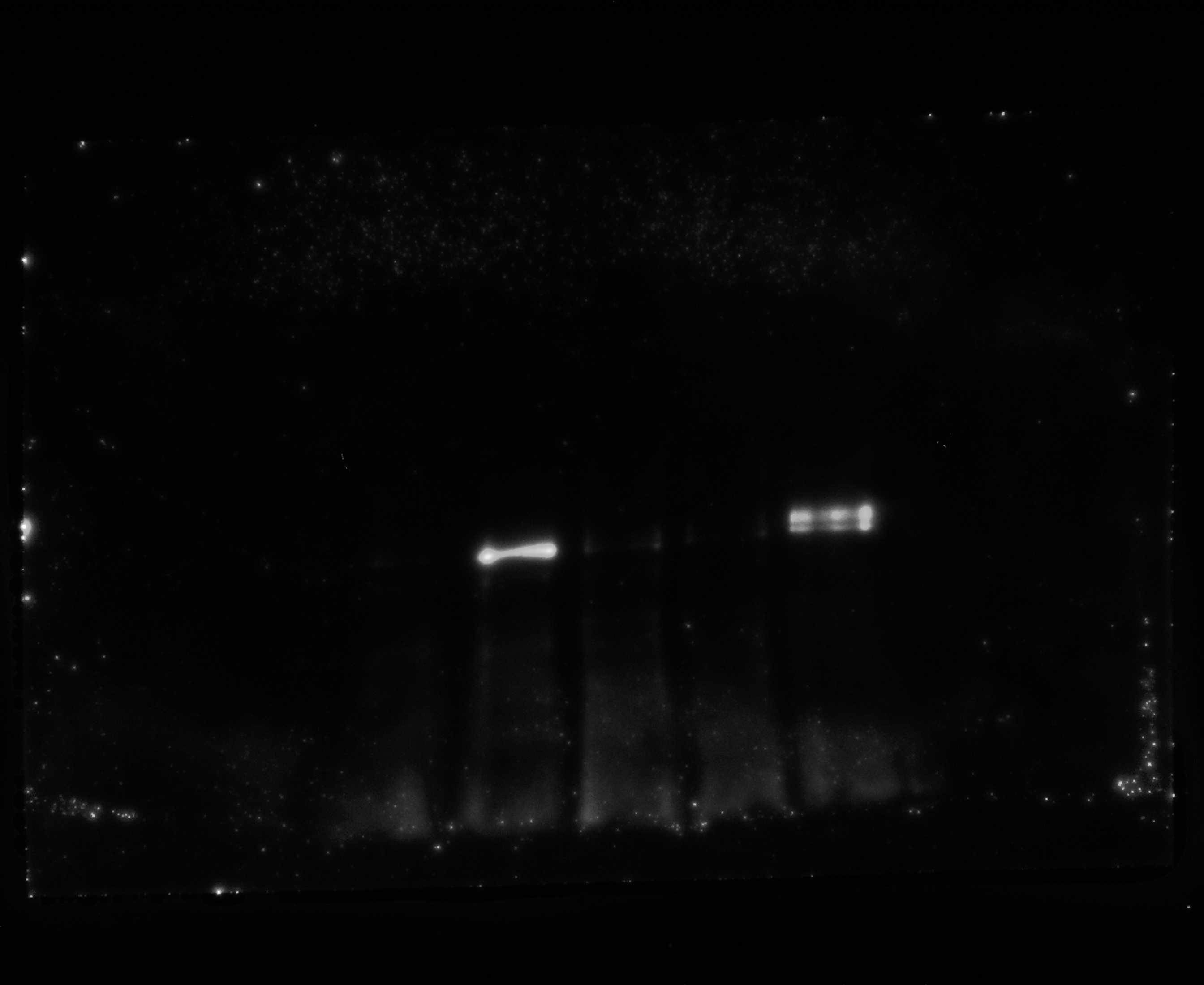

Supplement: Figure 2—source data 1. [file elife-92706-fig2-data1.zip › Figure 2 - source data 1_Original blots/Figure2A Scc1-3myc.Tif]

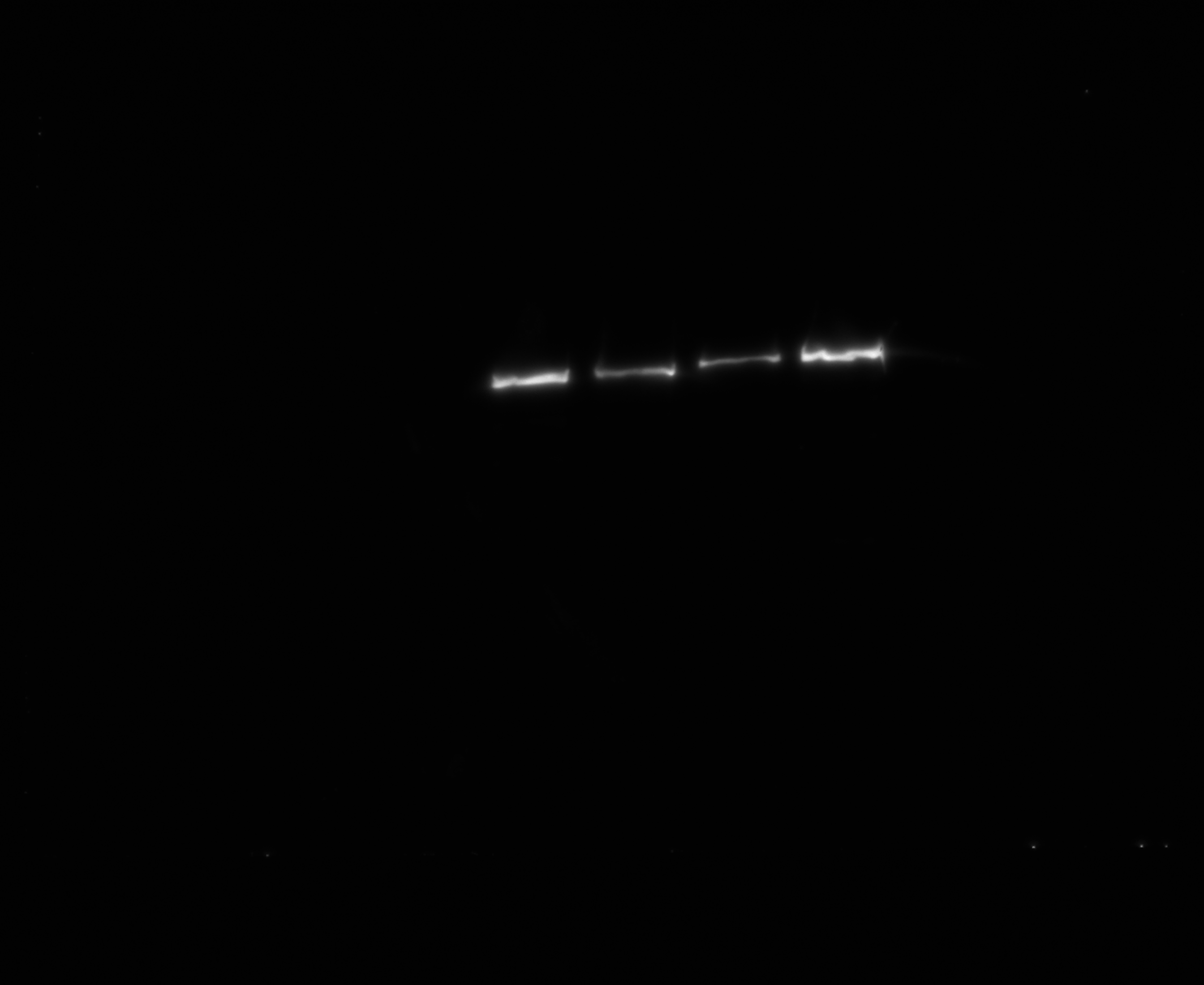

Supplement: Figure 2—source data 1. [file elife-92706-fig2-data1.zip › Figure 2 - source data 1_Original blots/Figure2A Smc1-6HA.Tif]

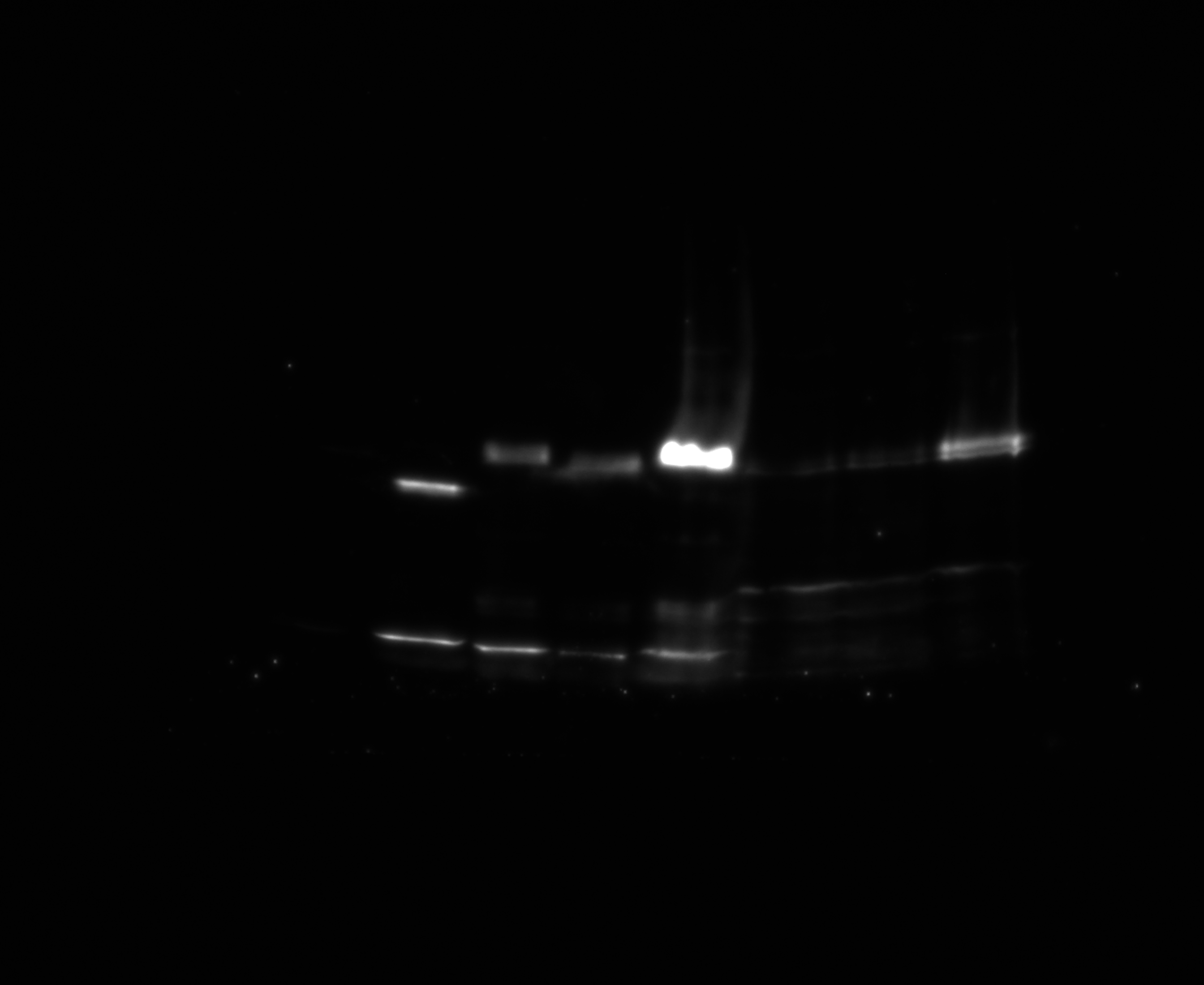

Supplement: Figure 2—source data 1. [file elife-92706-fig2-data1.zip › Figure 2 - source data 1_Original blots/Figure2B Chromatin_bound Scc1-3myc.Tif]

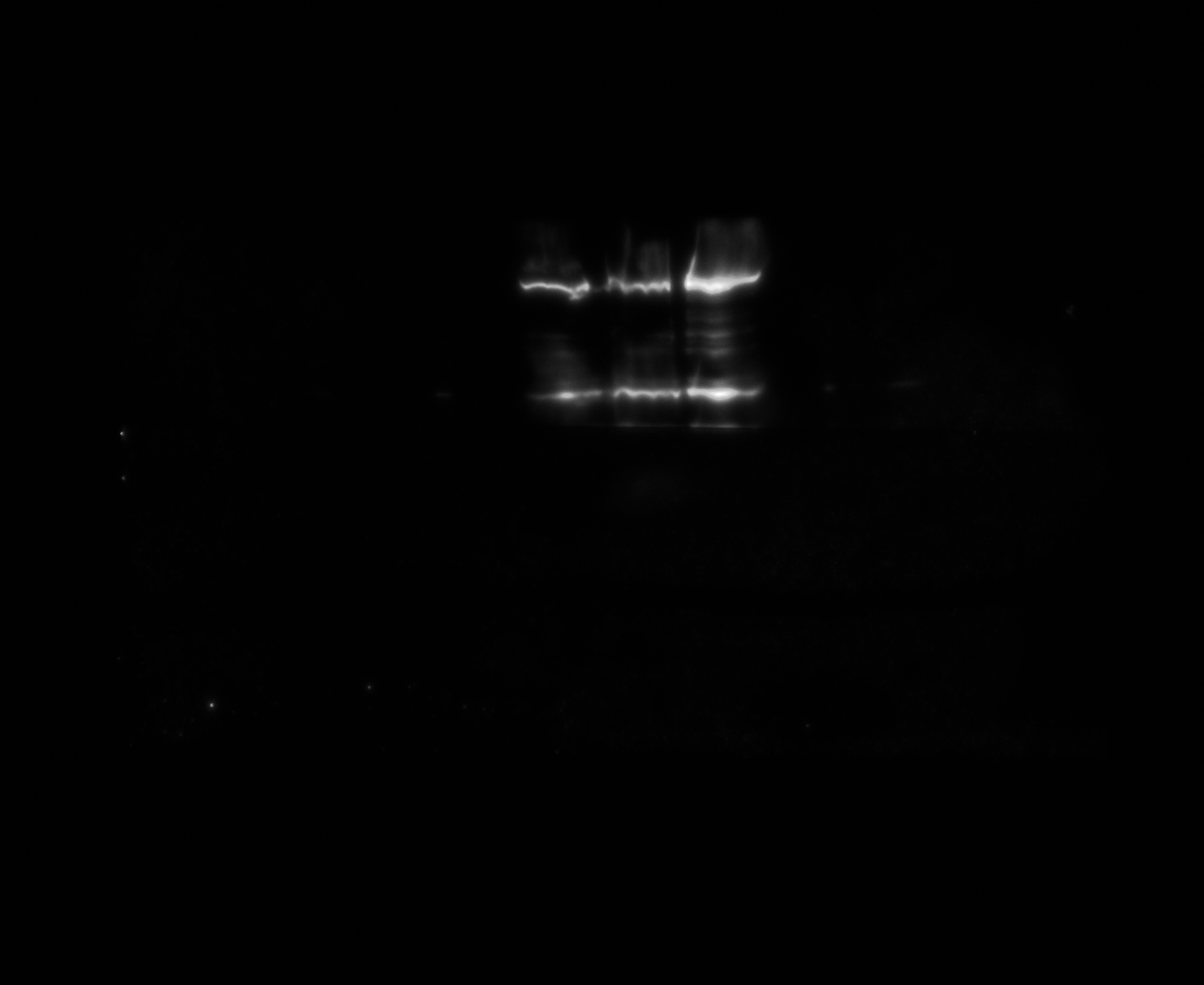

Supplement: Figure 2—source data 1. [file elife-92706-fig2-data1.zip › Figure 2 - source data 1_Original blots/Figure2B Chromatin_bound Smc1-6HA.Tif]

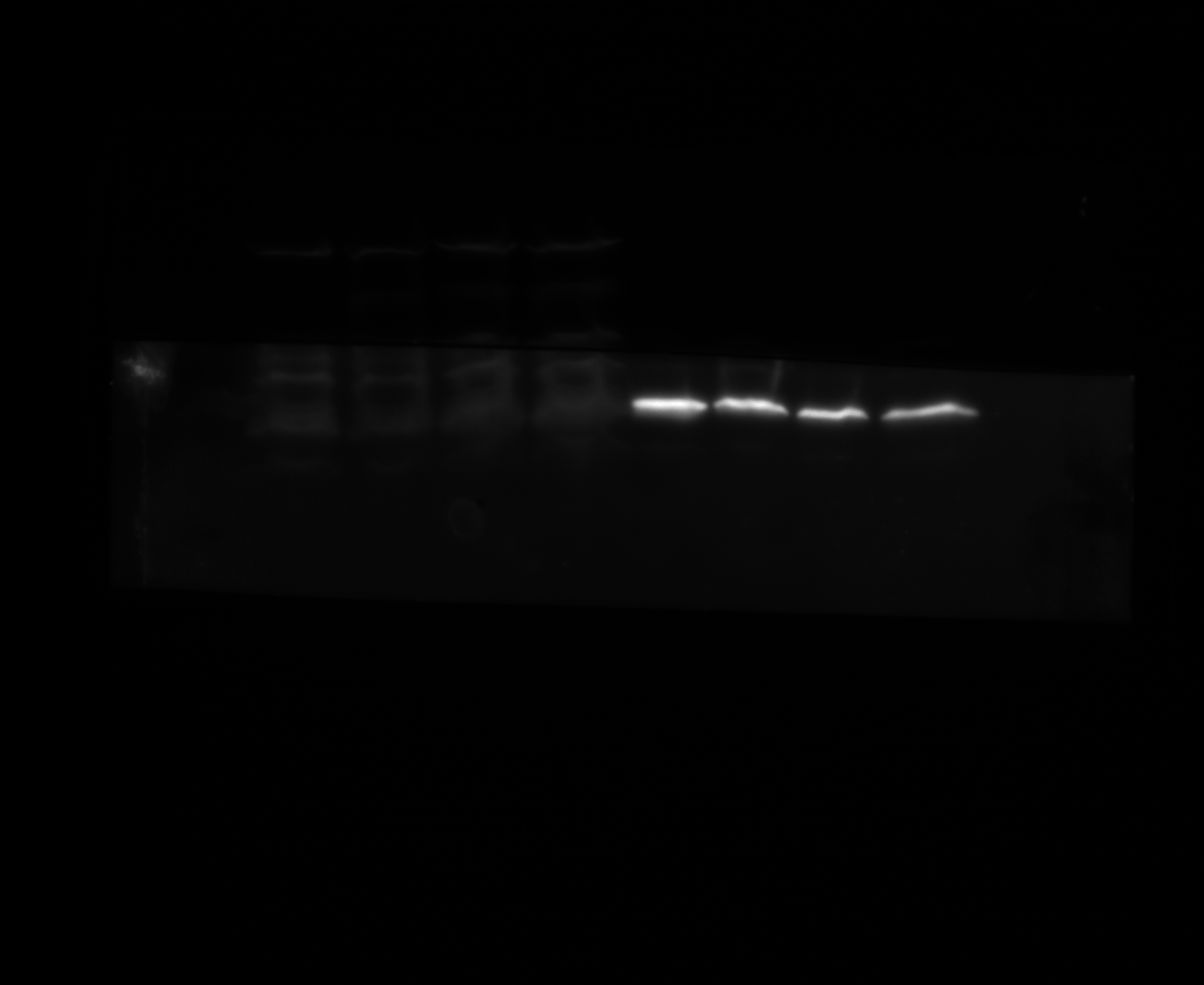

Supplement: Figure 2—source data 1. [file elife-92706-fig2-data1.zip › Figure 2 - source data 1_Original blots/Figure2B HH3.Tif]

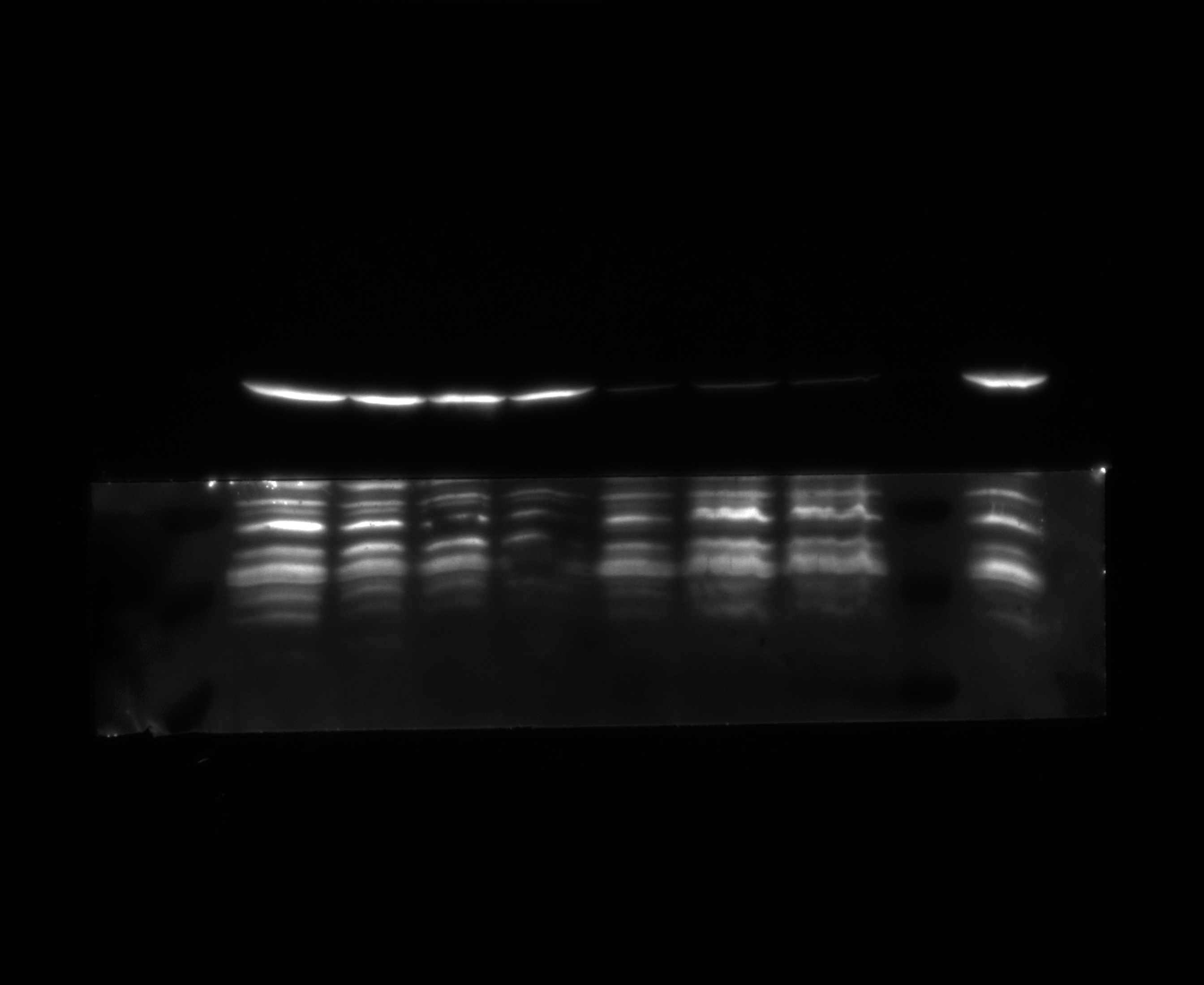

Supplement: Figure 2—source data 1. [file elife-92706-fig2-data1.zip › Figure 2 - source data 1_Original blots/Figure2B Pgk1.Tif]

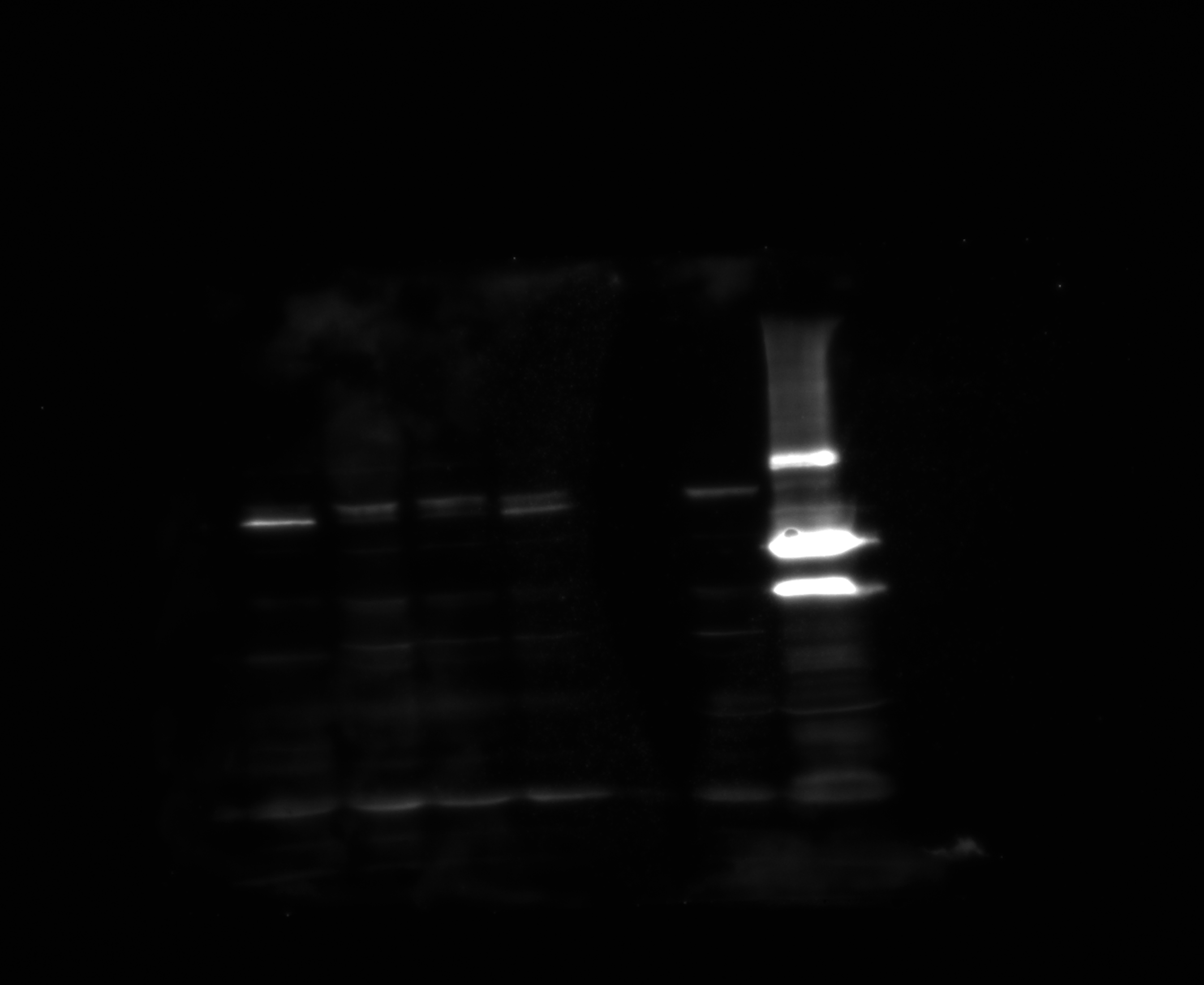

Supplement: Figure 2—source data 1. [file elife-92706-fig2-data1.zip › Figure 2 - source data 1_Original blots/Figure2B WCE Scc1-3myc.Tif]

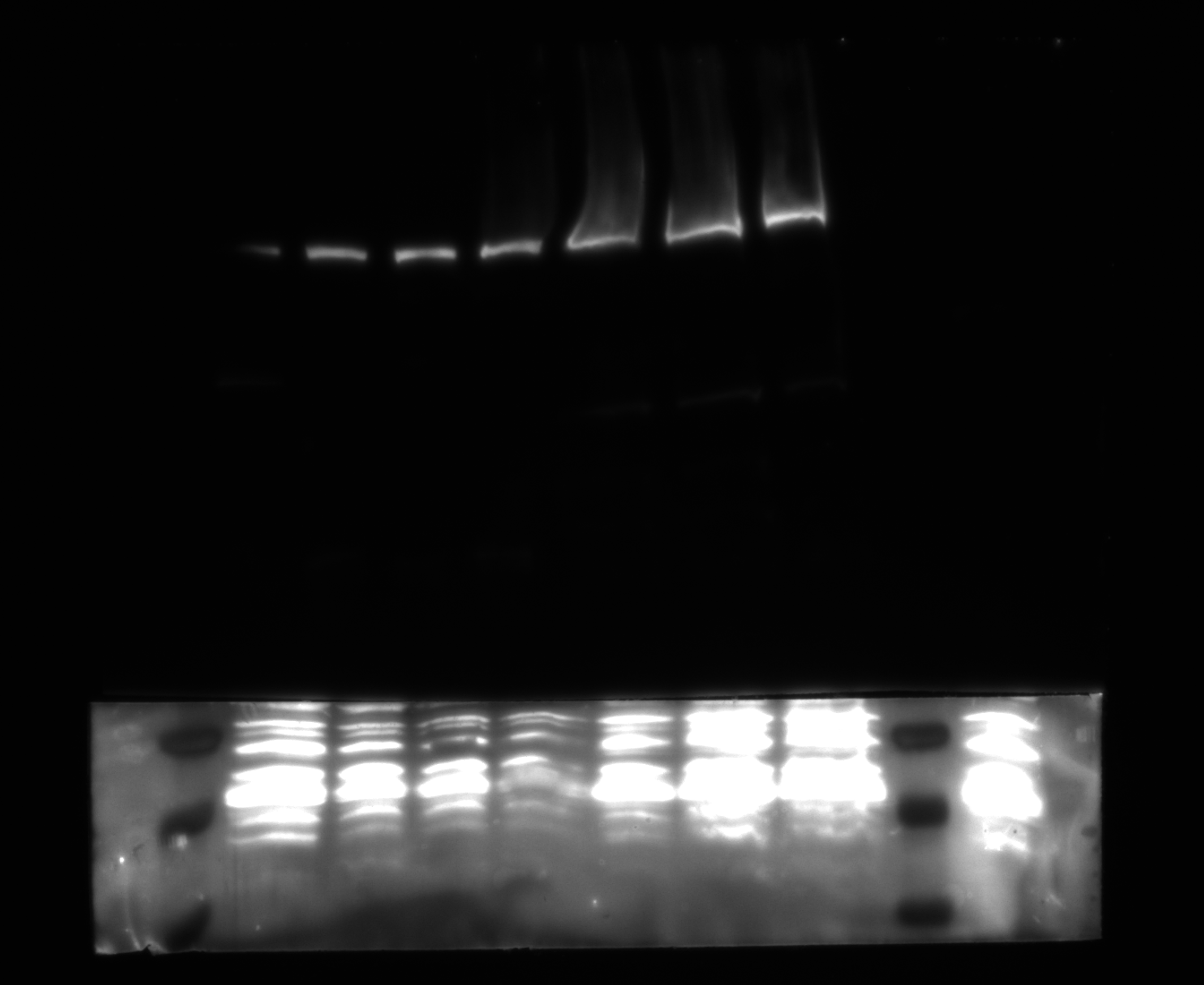

Supplement: Figure 2—source data 1. [file elife-92706-fig2-data1.zip › Figure 2 - source data 1_Original blots/Figure2B WCE Smc1-6HA.Tif]

Figure 2A

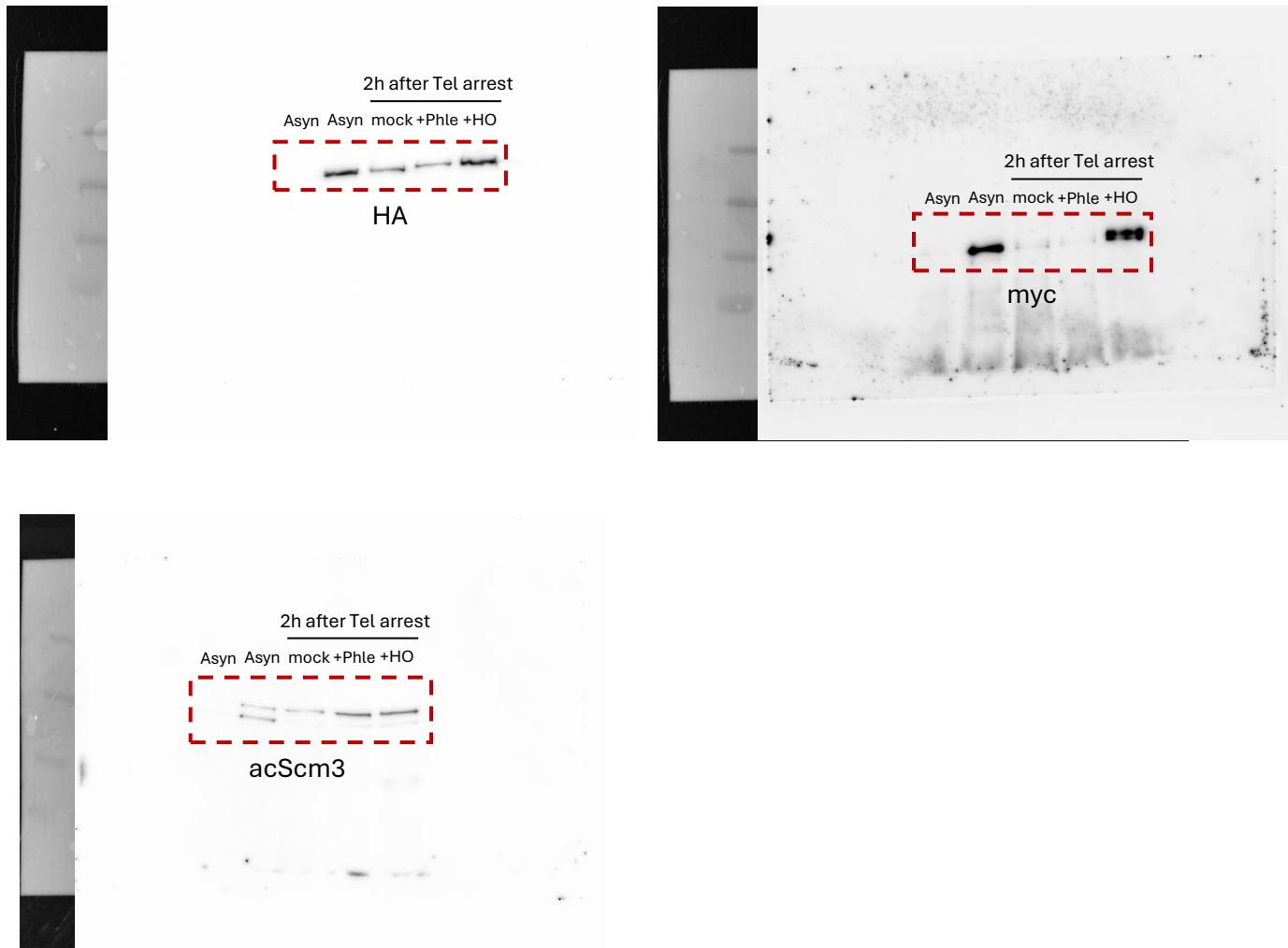

Figure 2B

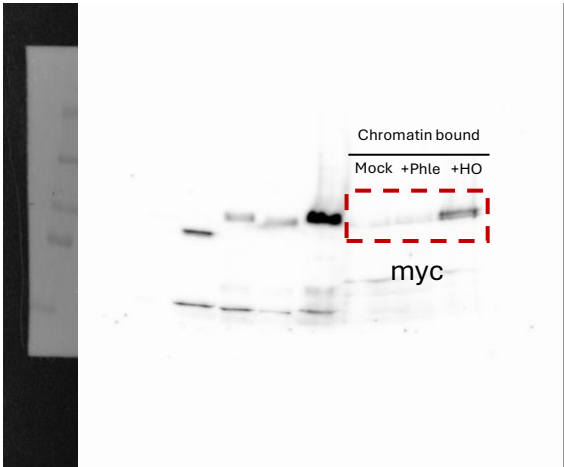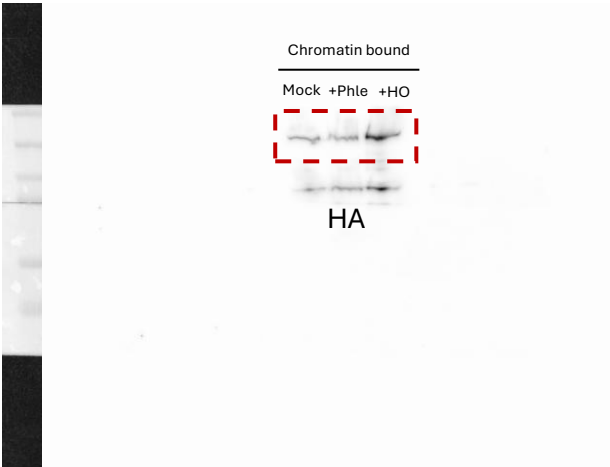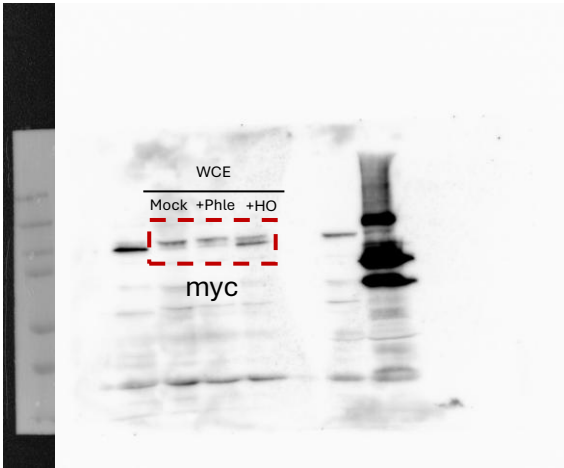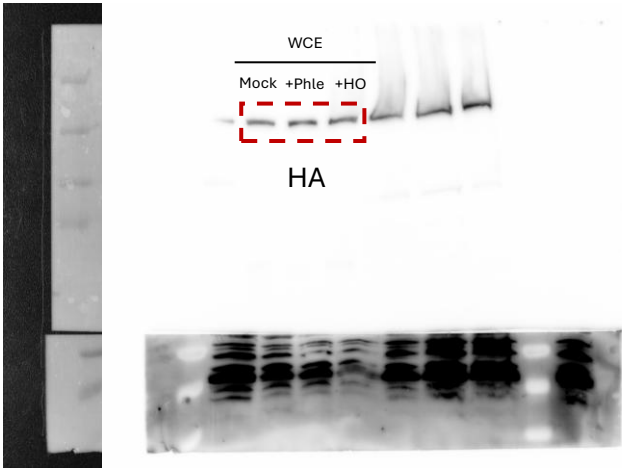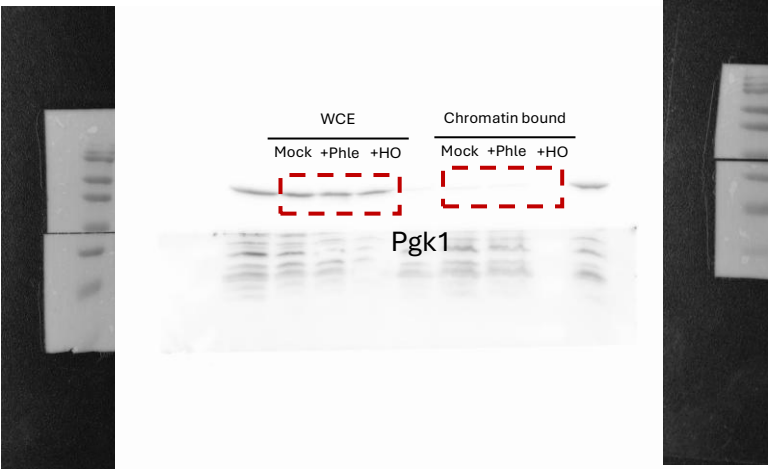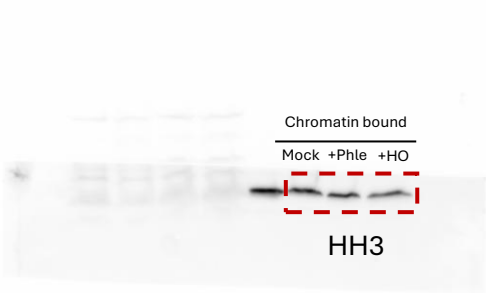

Supplement: Figure 2—source data 2. [file elife-92706-fig2-data2.pdf]

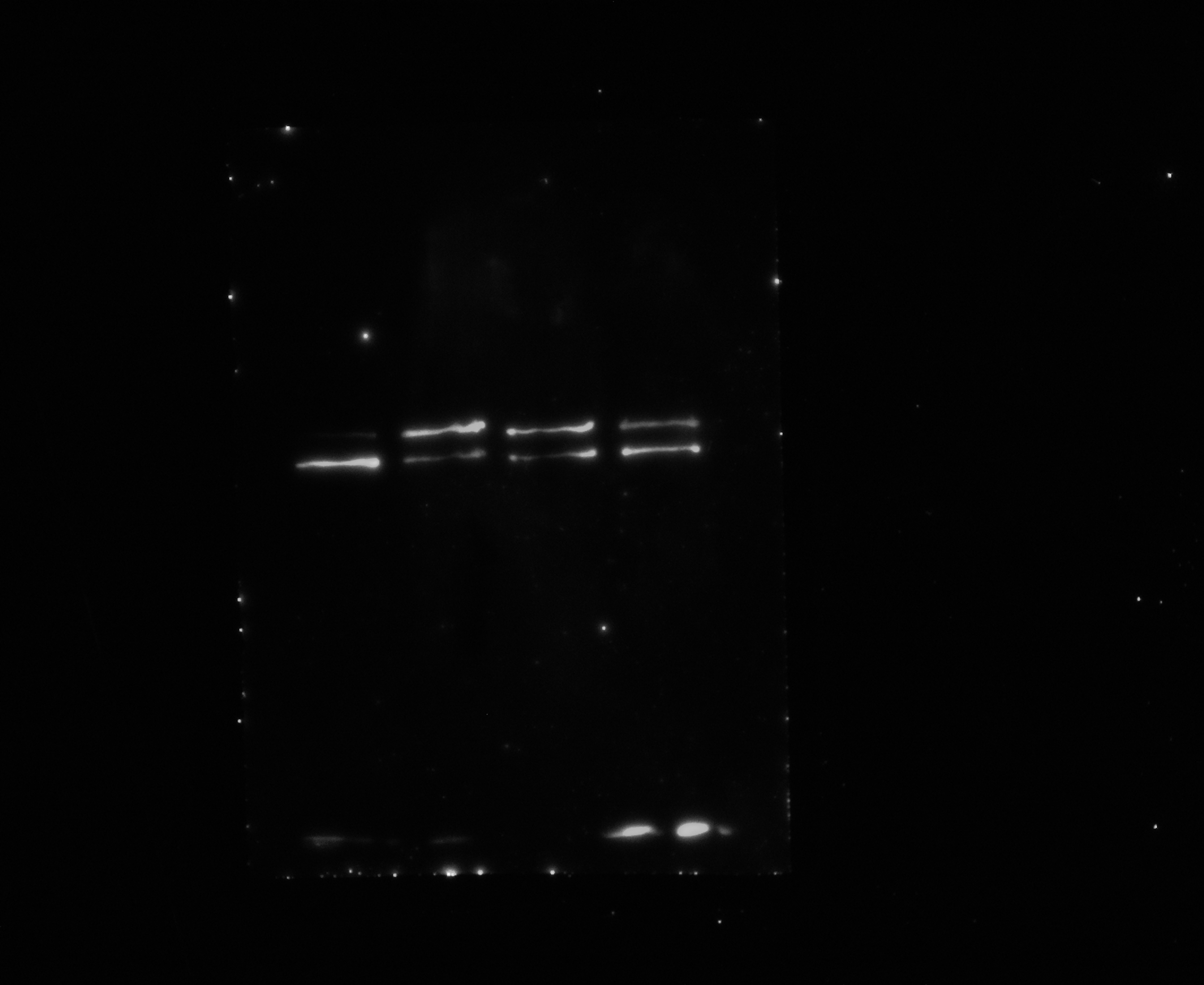

Supplement: Figure 2—figure supplement 1—source data 1. [file elife-92706-fig2-figsupp1-data1.zip › Figure 2 - figure supplement 1 - source data 1_original blots/Fig2-FS1 acScmc3.Tif]

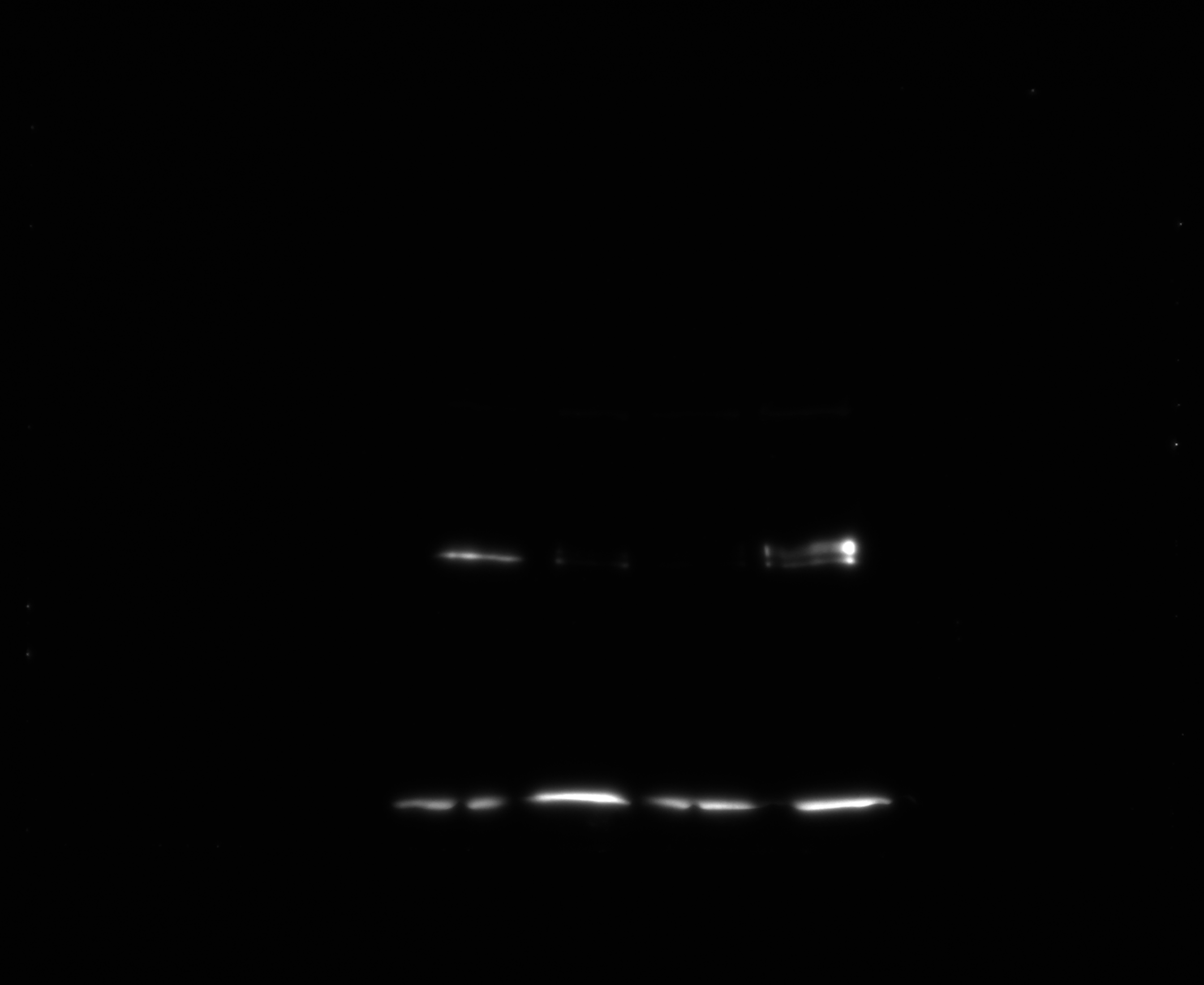

Supplement: Figure 2—figure supplement 1—source data 1. [file elife-92706-fig2-figsupp1-data1.zip › Figure 2 - figure supplement 1 - source data 1_original blots/Fig2-FS1 Scc1-3myc.Tif]

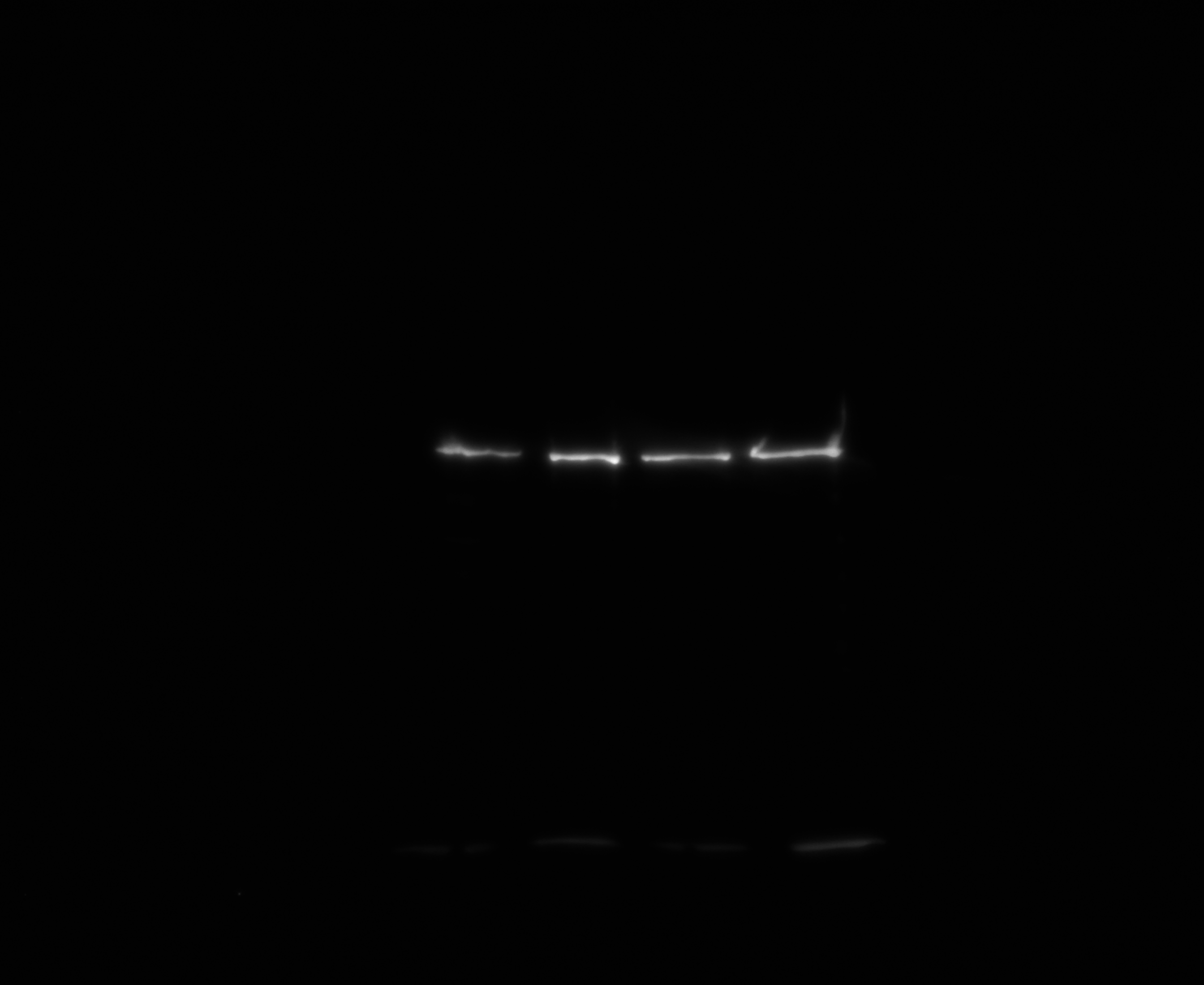

Supplement: Figure 2—figure supplement 1—source data 1. [file elife-92706-fig2-figsupp1-data1.zip › Figure 2 - figure supplement 1 - source data 1_original blots/Fig2-FS1 Smc1-6HA.Tif]

Figure S1

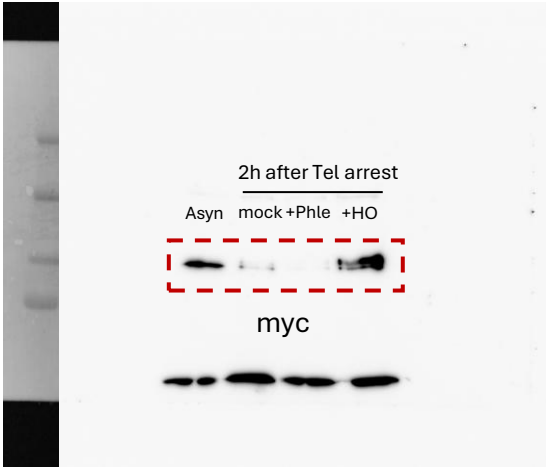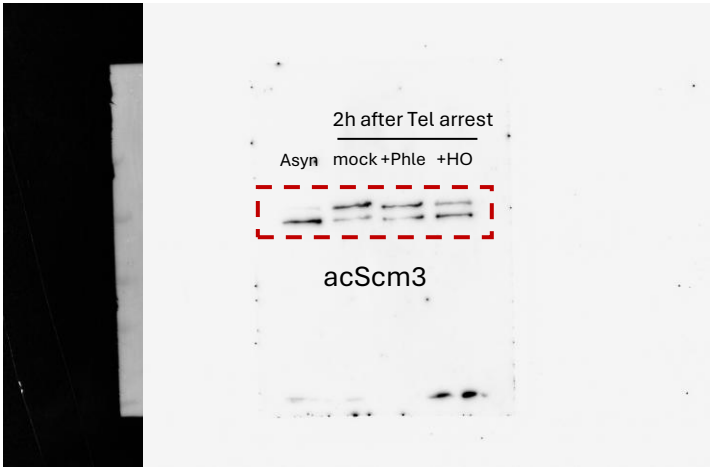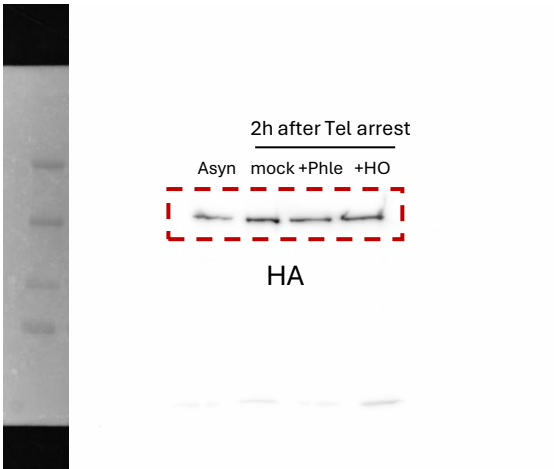

Supplement: Figure 2—figure supplement 1—source data 2. [file elife-92706-fig2-figsupp1-data2.pdf]

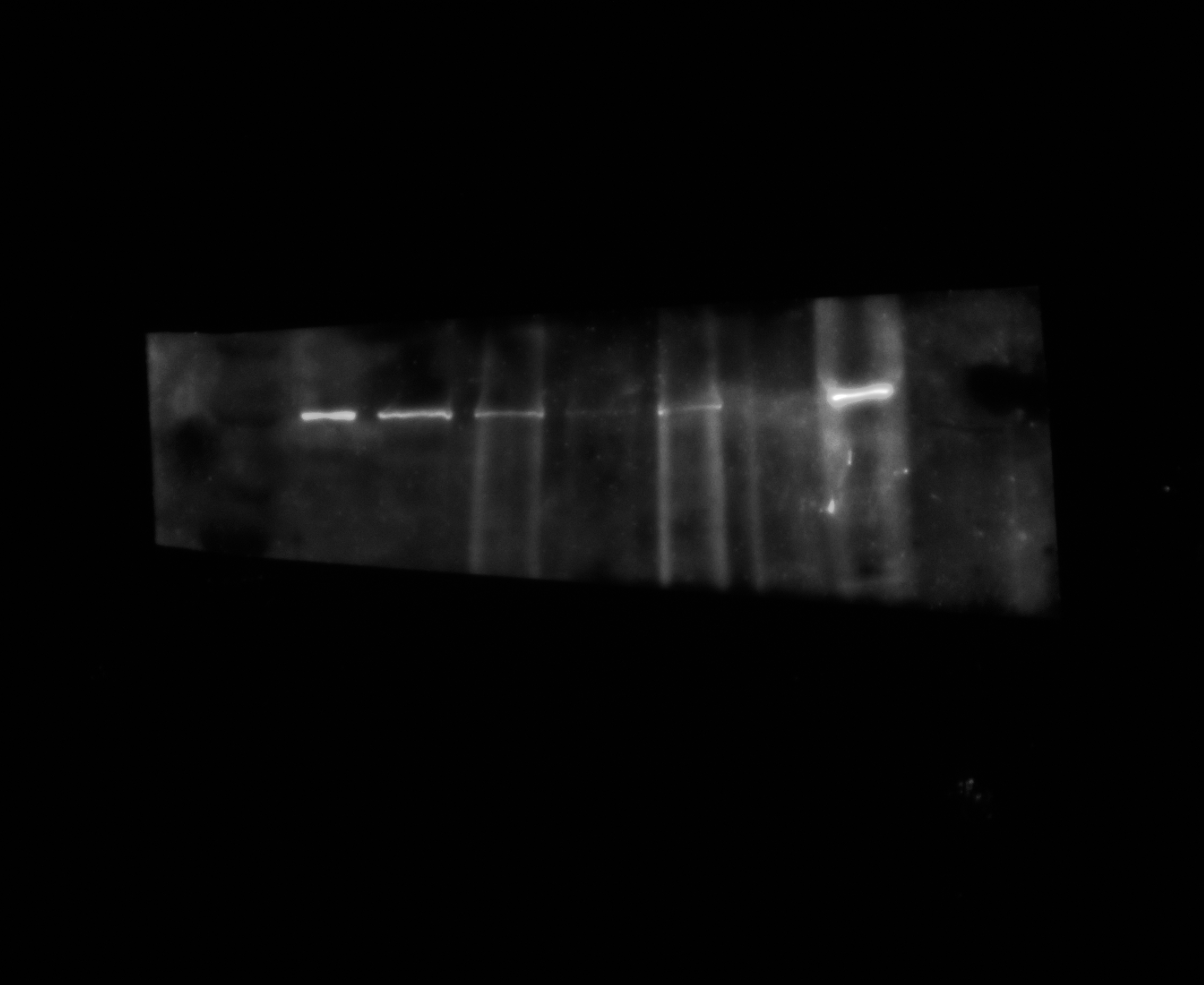

Supplement: Figure 2—figure supplement 2—source data 1. [file elife-92706-fig2-figsupp2-data1.zip › Figure 2 - figure supplement 2 - source data 1_original blots/Fig2-FS2A acScm3.Tif]

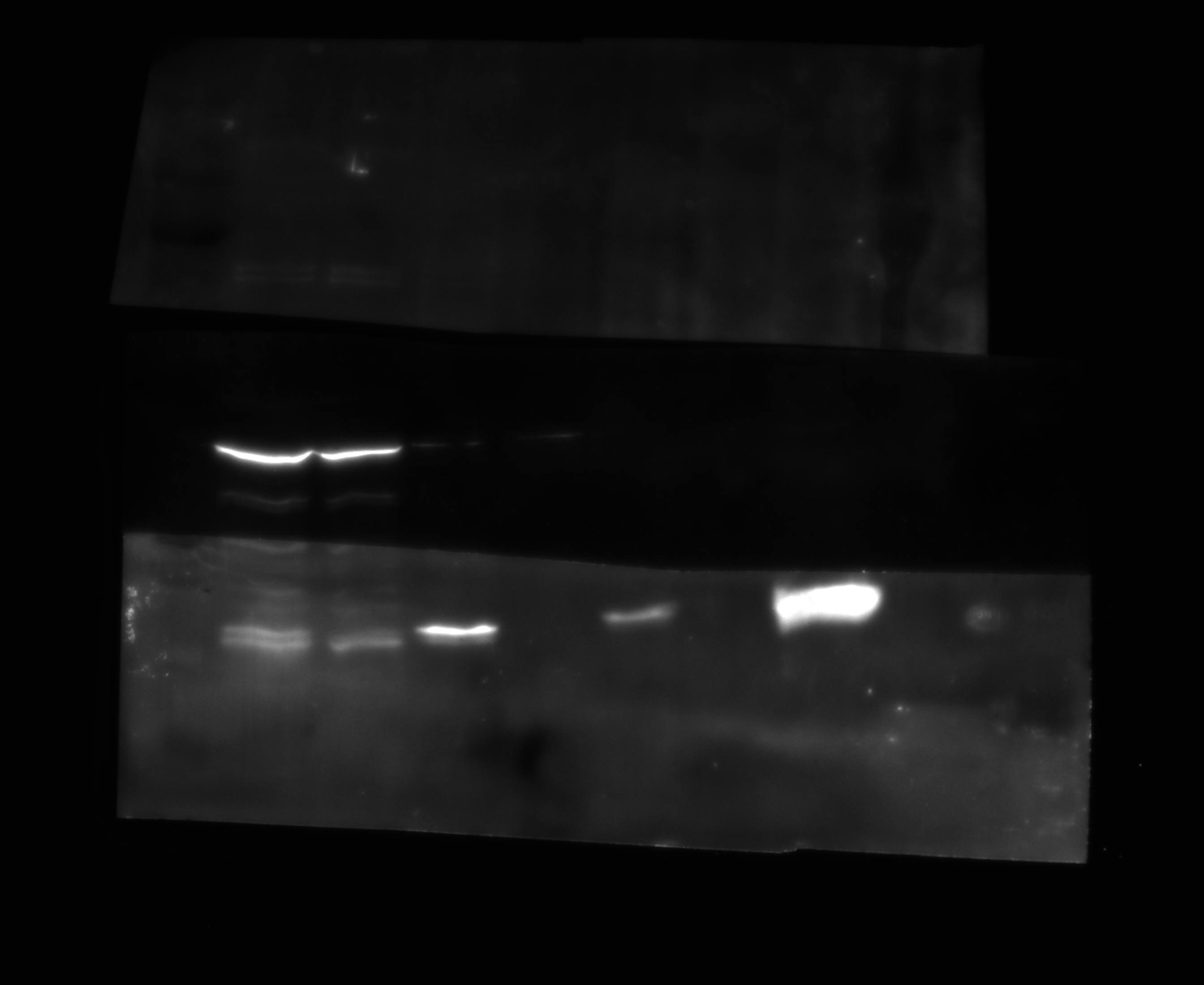

Supplement: Figure 2—figure supplement 2—source data 1. [file elife-92706-fig2-figsupp2-data1.zip › Figure 2 - figure supplement 2 - source data 1_original blots/Fig2-FS2A HH3.Tif]

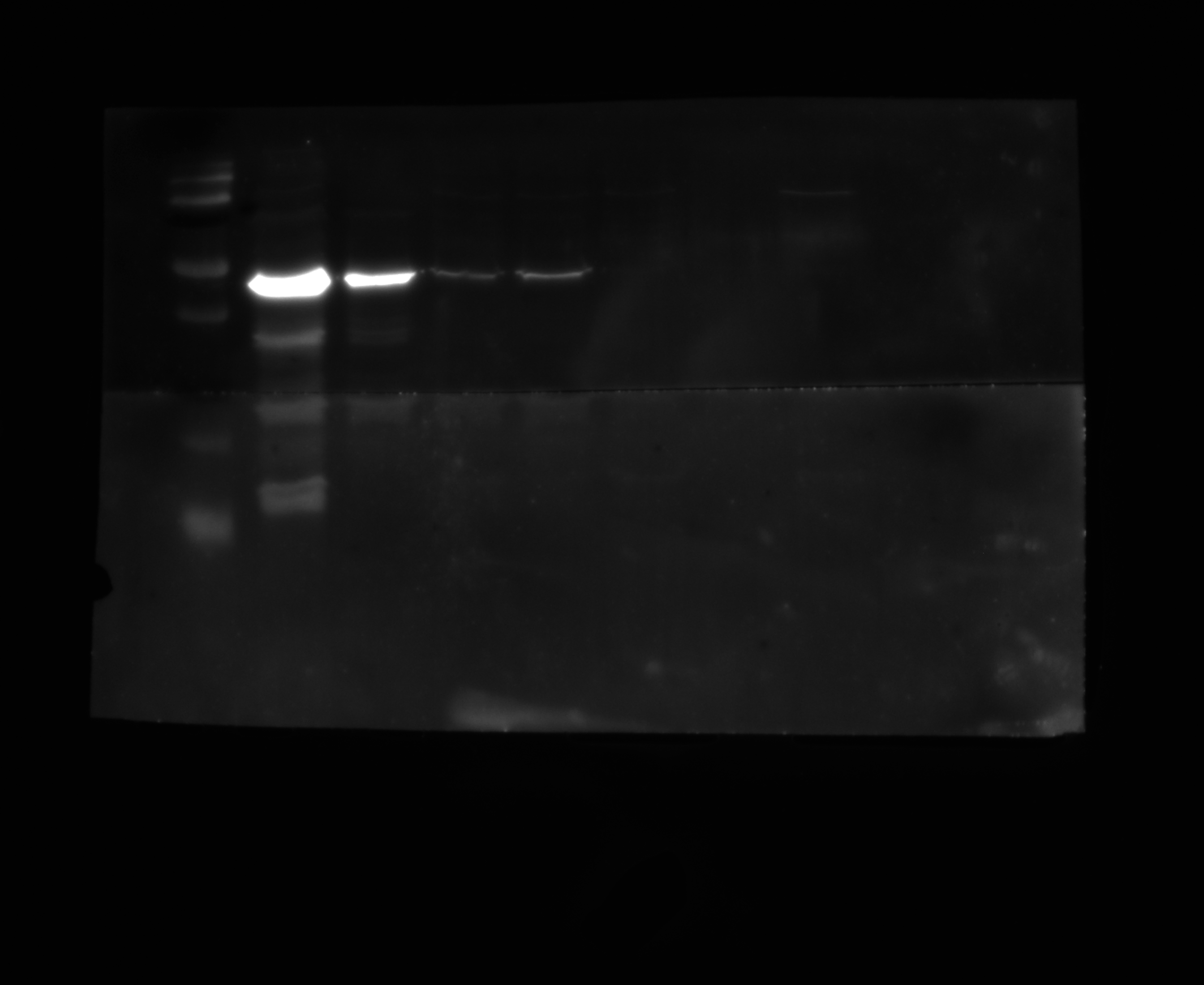

Supplement: Figure 2—figure supplement 2—source data 1. [file elife-92706-fig2-figsupp2-data1.zip › Figure 2 - figure supplement 2 - source data 1_original blots/Fig2-FS2A Pgk1.Tif]

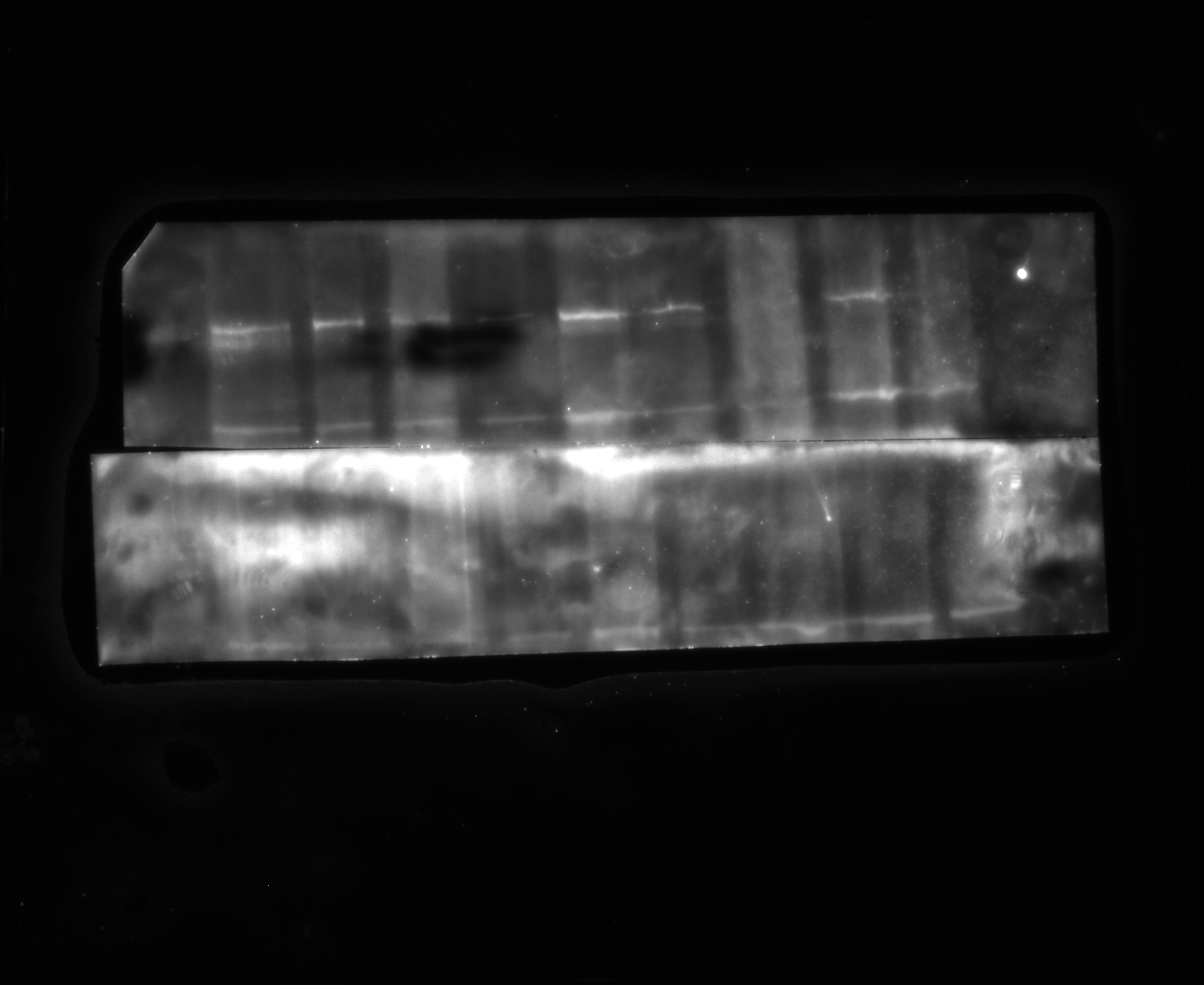

Supplement: Figure 2—figure supplement 2—source data 1. [file elife-92706-fig2-figsupp2-data1.zip › Figure 2 - figure supplement 2 - source data 1_original blots/Fig2-FS2B Chromatin bound acScm3.Tif]

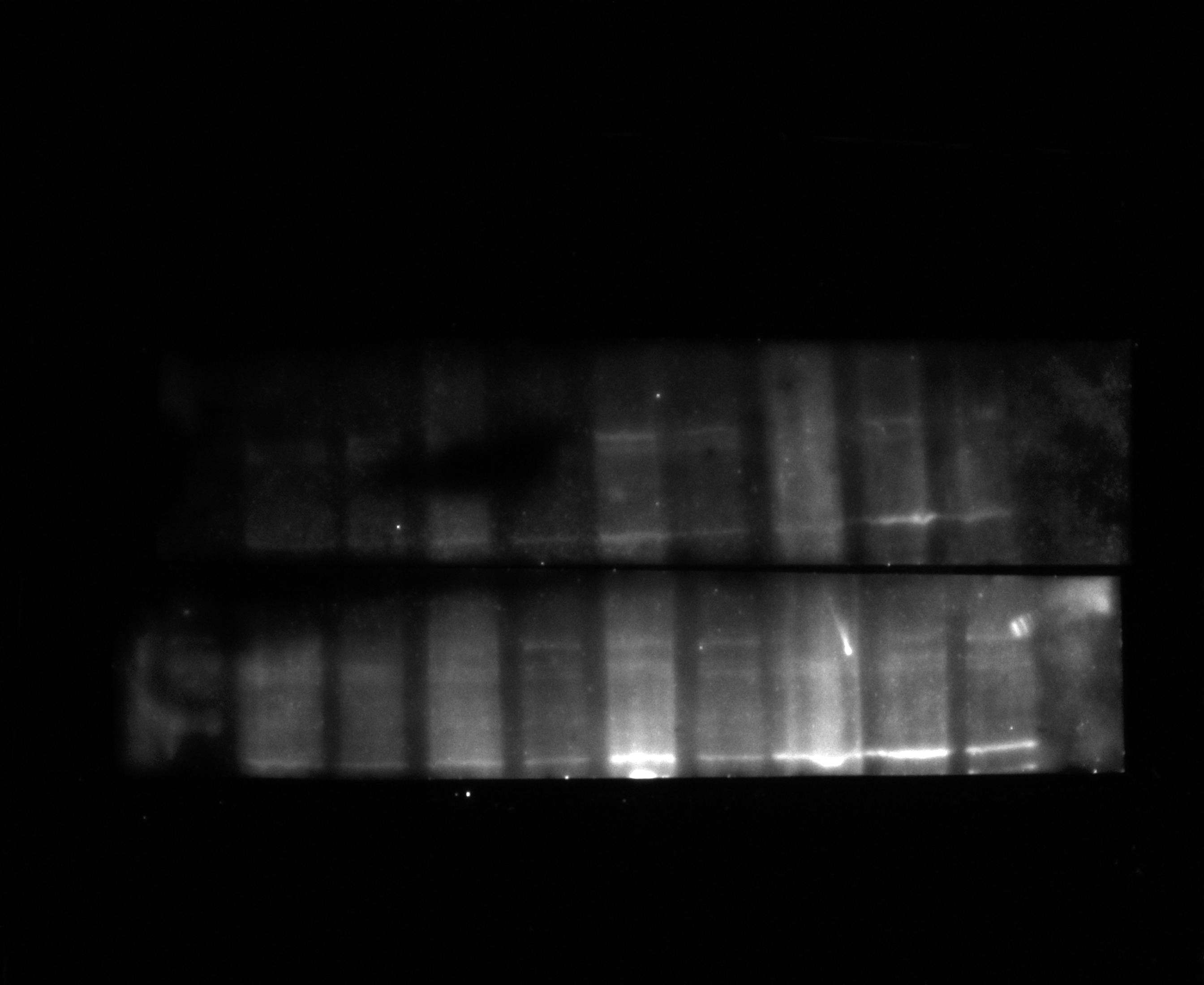

Supplement: Figure 2—figure supplement 2—source data 1. [file elife-92706-fig2-figsupp2-data1.zip › Figure 2 - figure supplement 2 - source data 1_original blots/Fig2-FS2B Chromatin bound Smc3-HA.Tif]

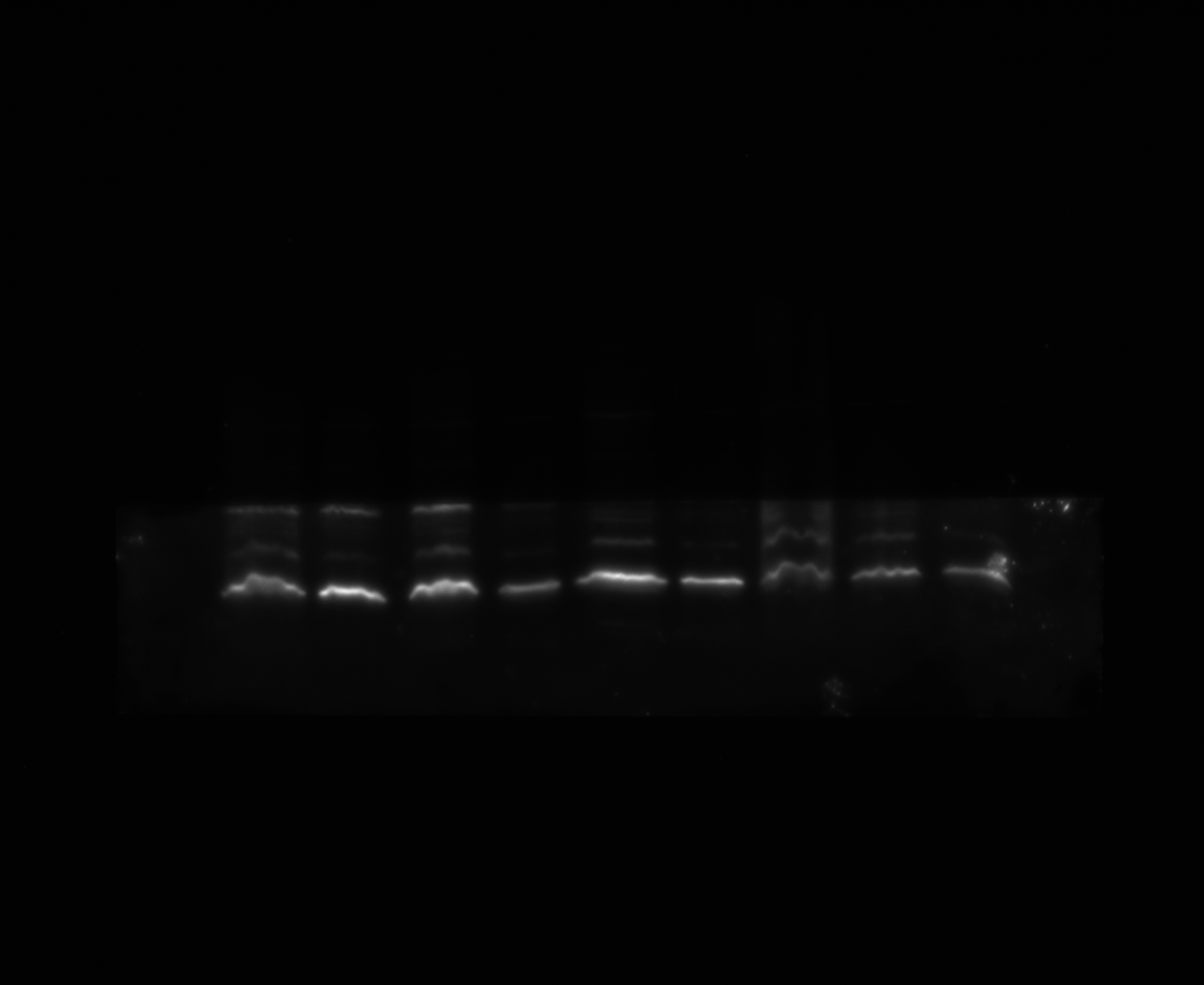

Supplement: Figure 2—figure supplement 2—source data 1. [file elife-92706-fig2-figsupp2-data1.zip › Figure 2 - figure supplement 2 - source data 1_original blots/Fig2-FS2B HH3.Tif]

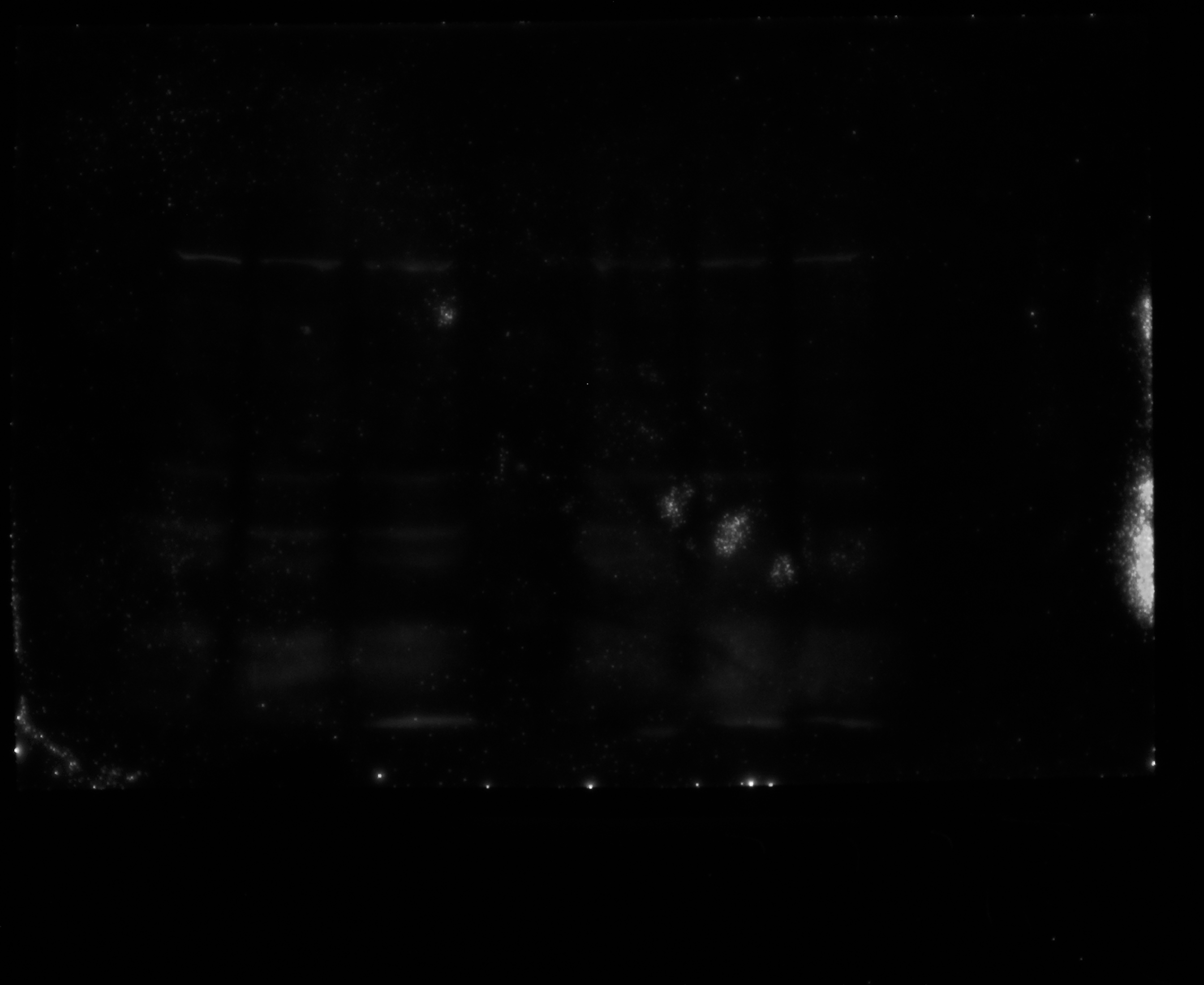

Supplement: Figure 2—figure supplement 2—source data 1. [file elife-92706-fig2-figsupp2-data1.zip › Figure 2 - figure supplement 2 - source data 1_original blots/Fig2-FS2B WCE acScm3.Tif]

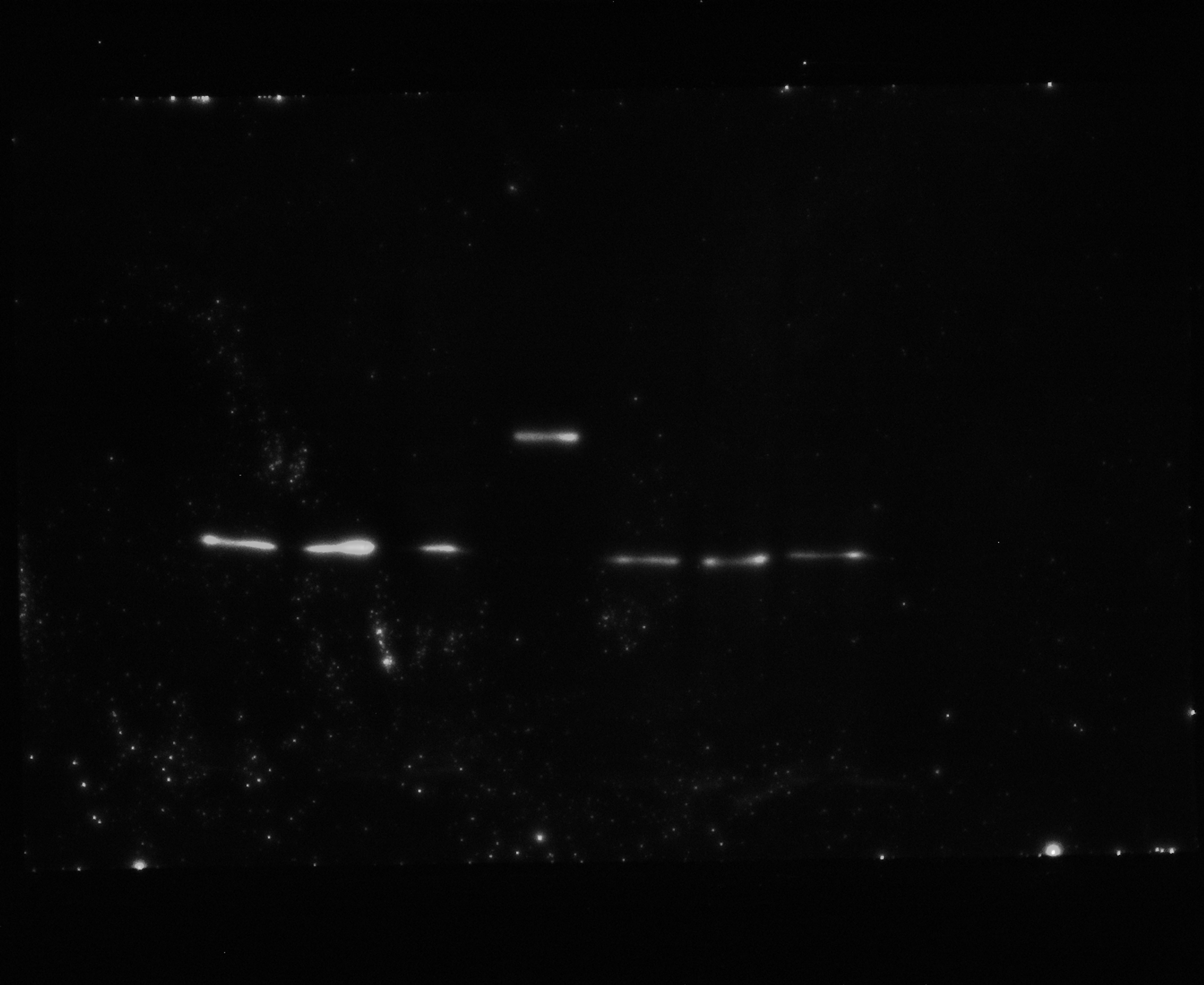

Supplement: Figure 2—figure supplement 2—source data 1. [file elife-92706-fig2-figsupp2-data1.zip › Figure 2 - figure supplement 2 - source data 1_original blots/Fig2-FS2B WCE Smc3-HA.Tif]

Figure S2A

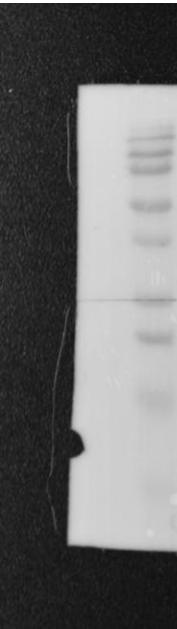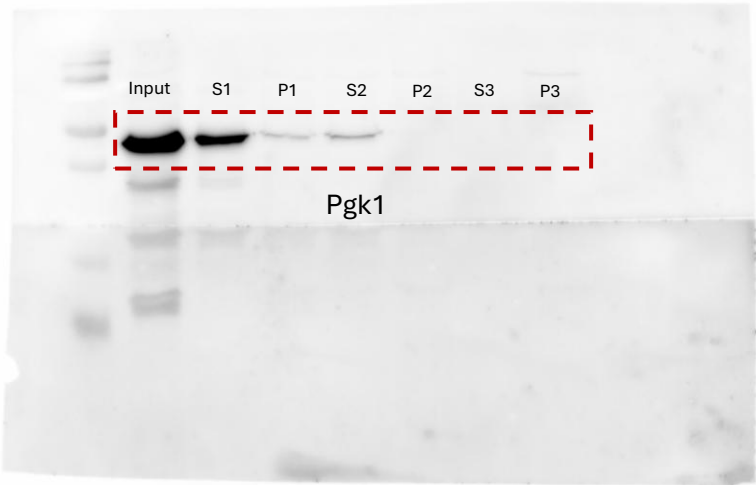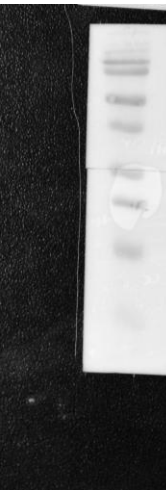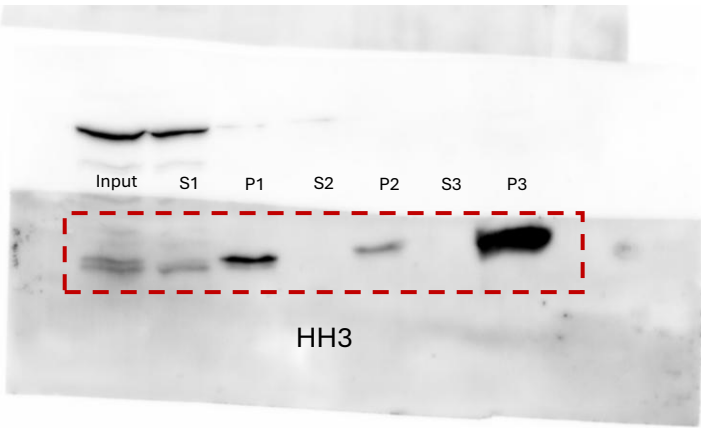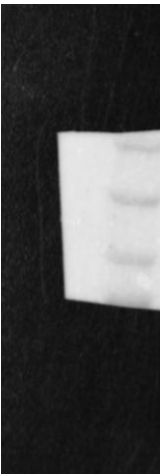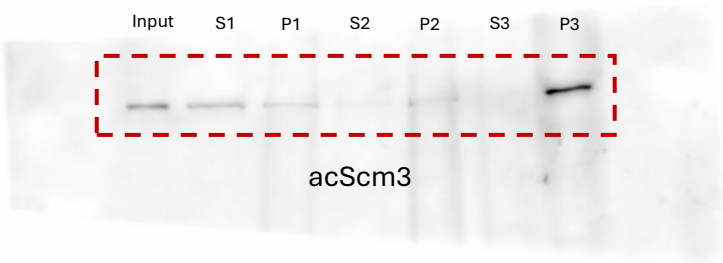

Figure S2B

Mock +HO +Phle

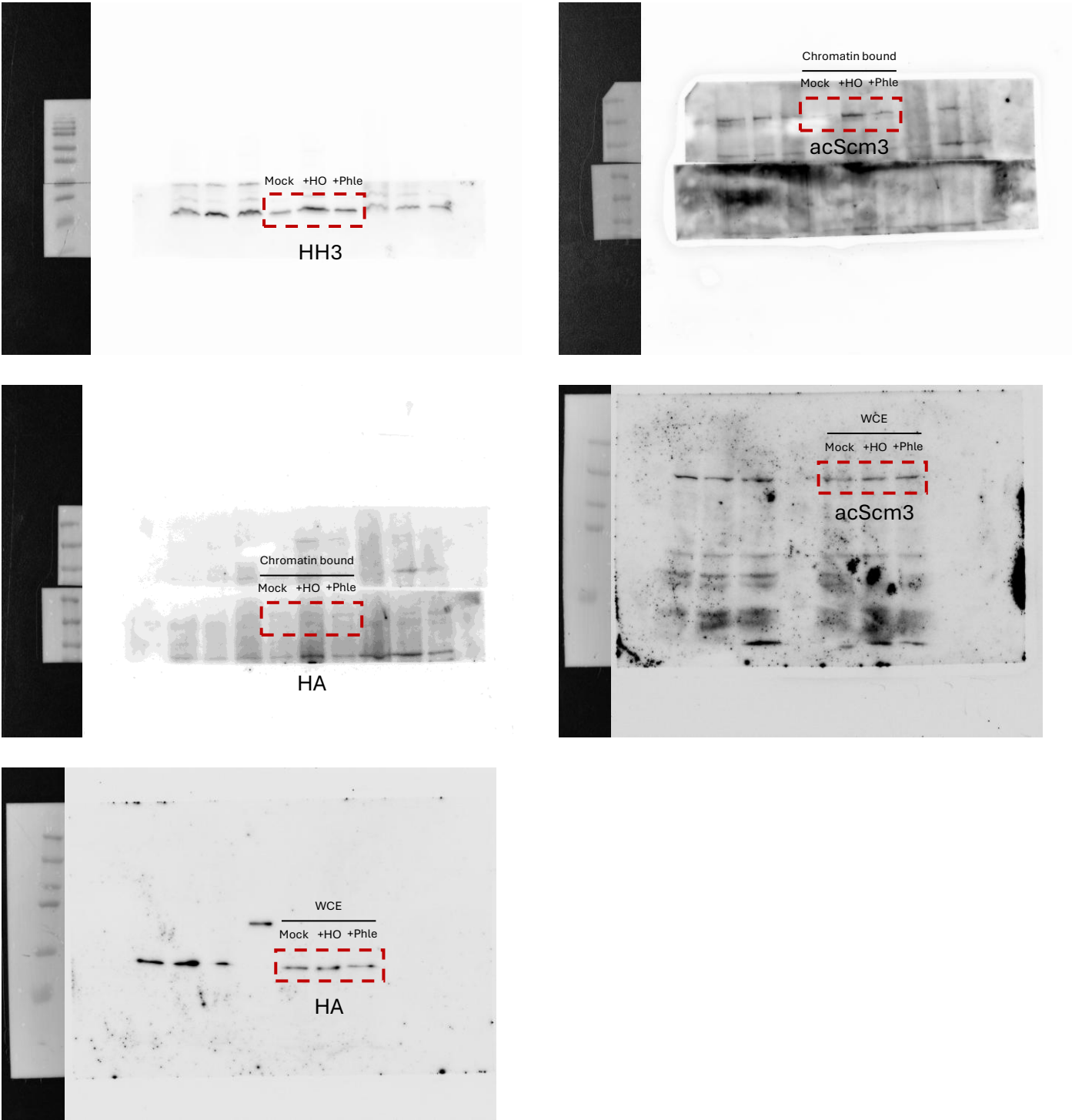

Supplement: Figure 2—figure supplement 2—source data 2. [file elife-92706-fig2-figsupp2-data2.pdf]

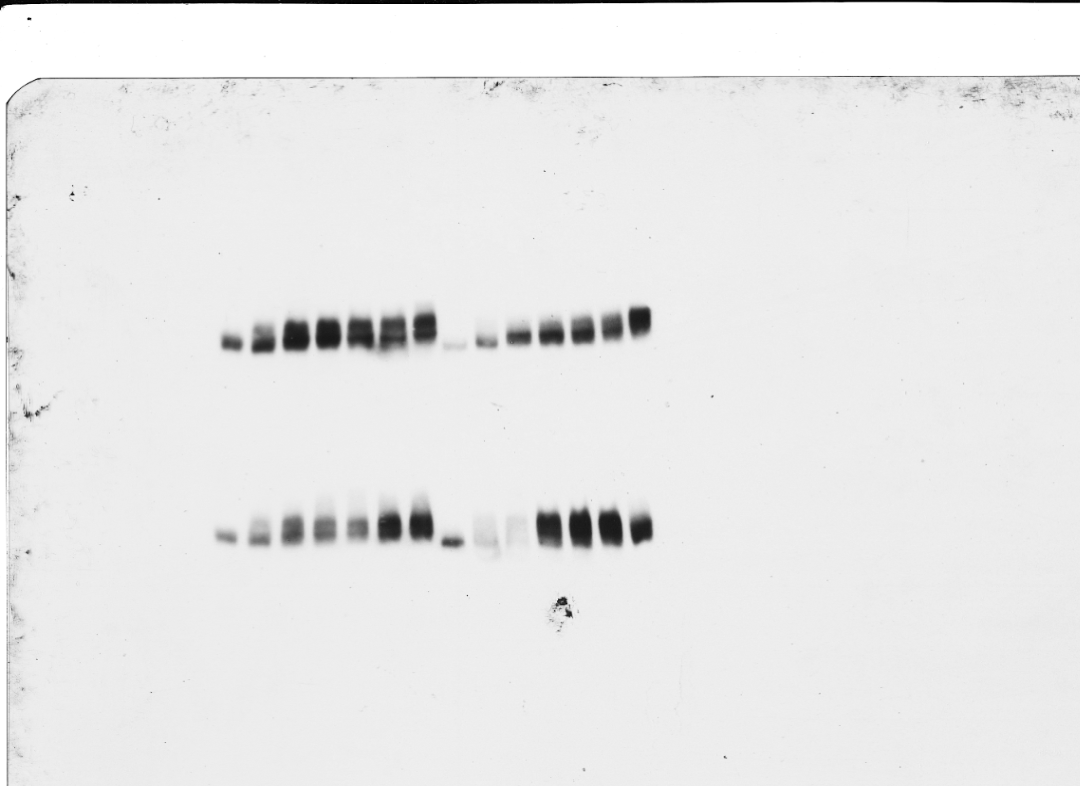

Supplement: Figure 3—source data 1. [file elife-92706-fig3-data1.zip › Figure 3 - source data 1_Original blots/Fig 3B_Rad53.tiff]

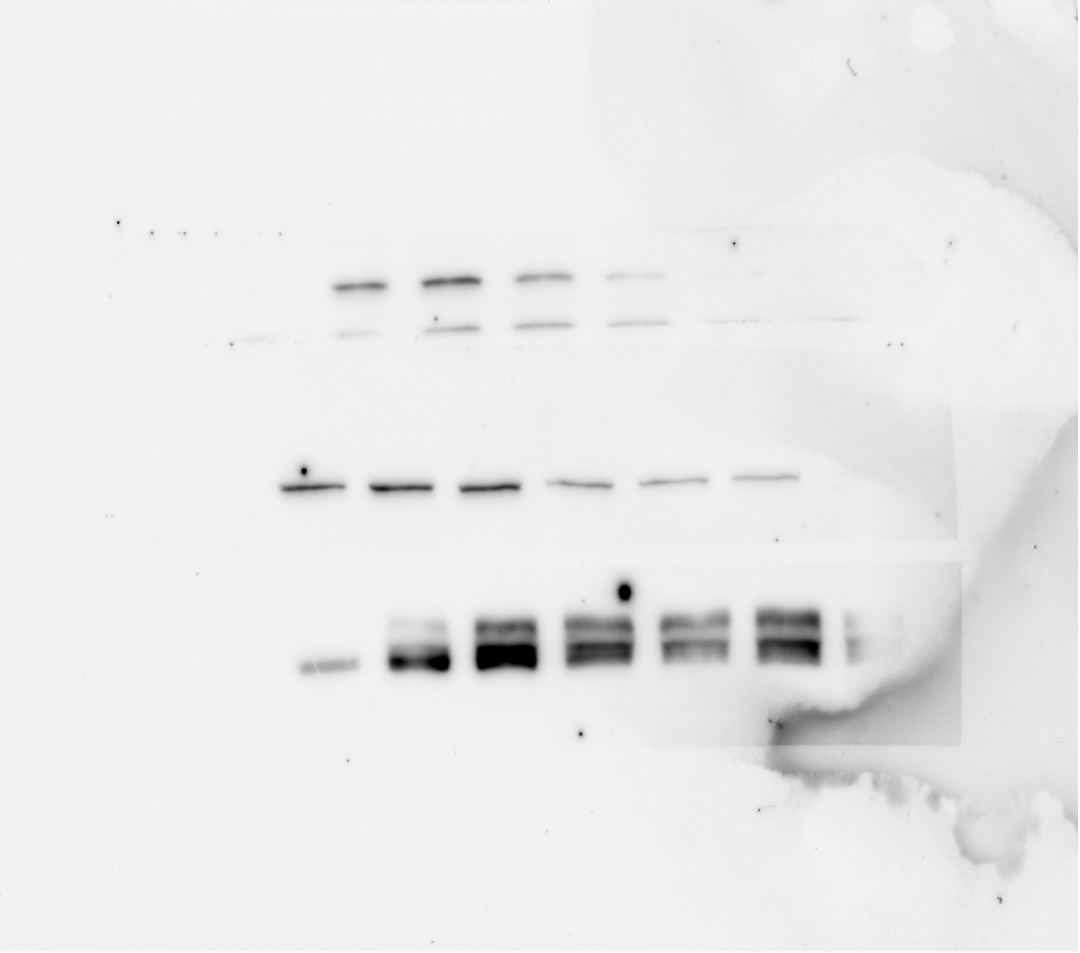

Supplement: Figure 3—source data 1. [file elife-92706-fig3-data1.zip › Figure 3 - source data 1_Original blots/Fig 3D_HO-Flag and Rad53.tiff]

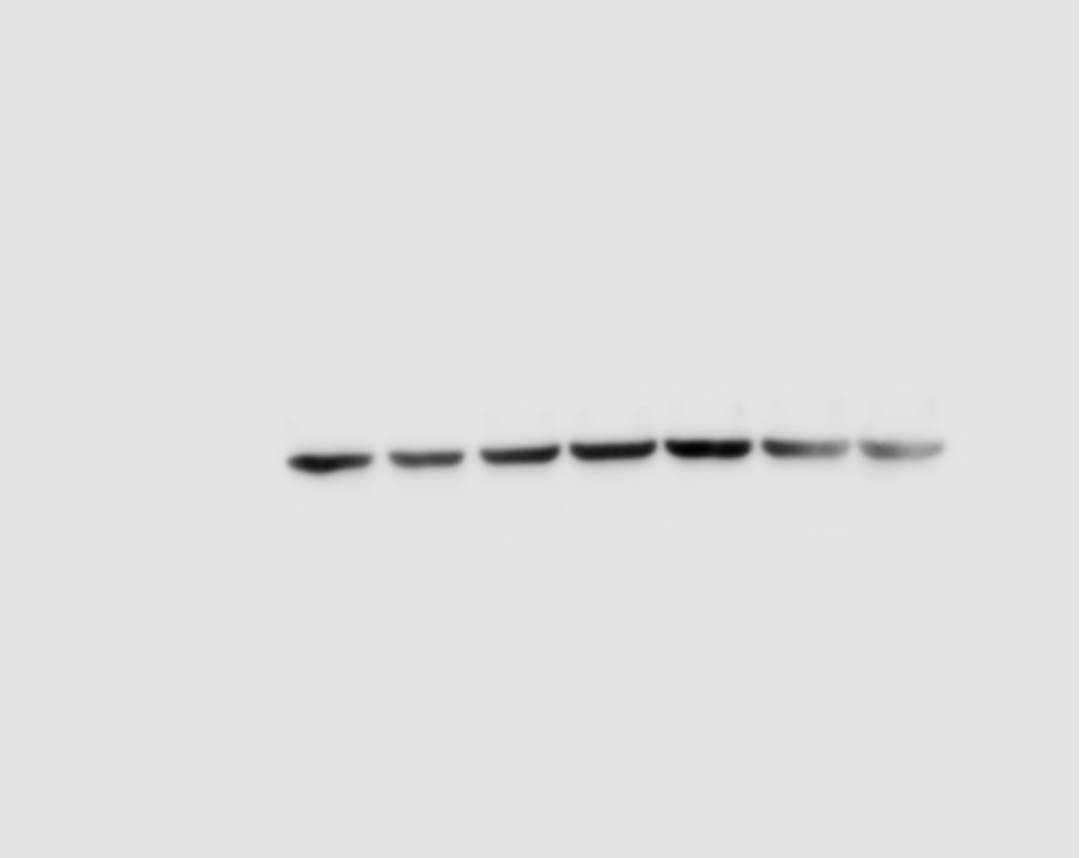

Supplement: Figure 3—source data 1. [file elife-92706-fig3-data1.zip › Figure 3 - source data 1_Original blots/Fig 3D_PGK1.tiff]

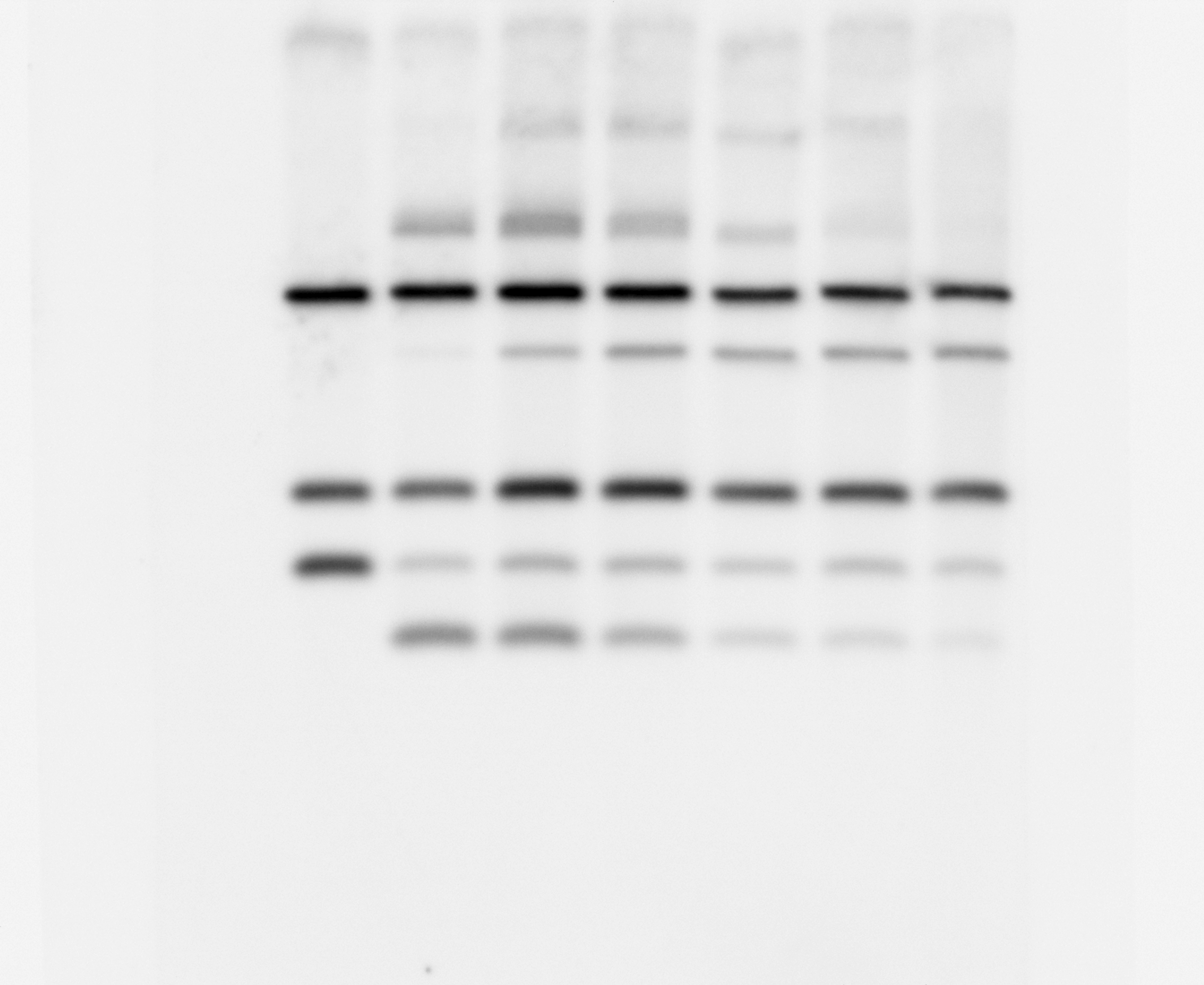

Supplement: Figure 3—source data 1. [file elife-92706-fig3-data1.zip › Figure 3 - source data 1_Original blots/Fig 3E_Southern blot.TIF]

Figure 3b – Western blots

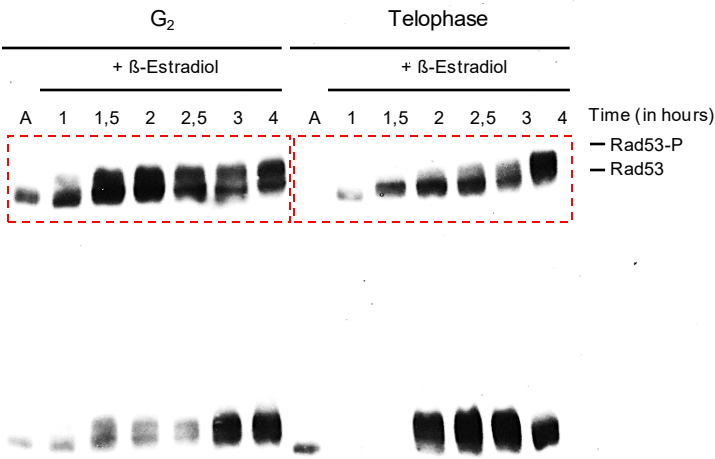

Figure 3d – Western blots

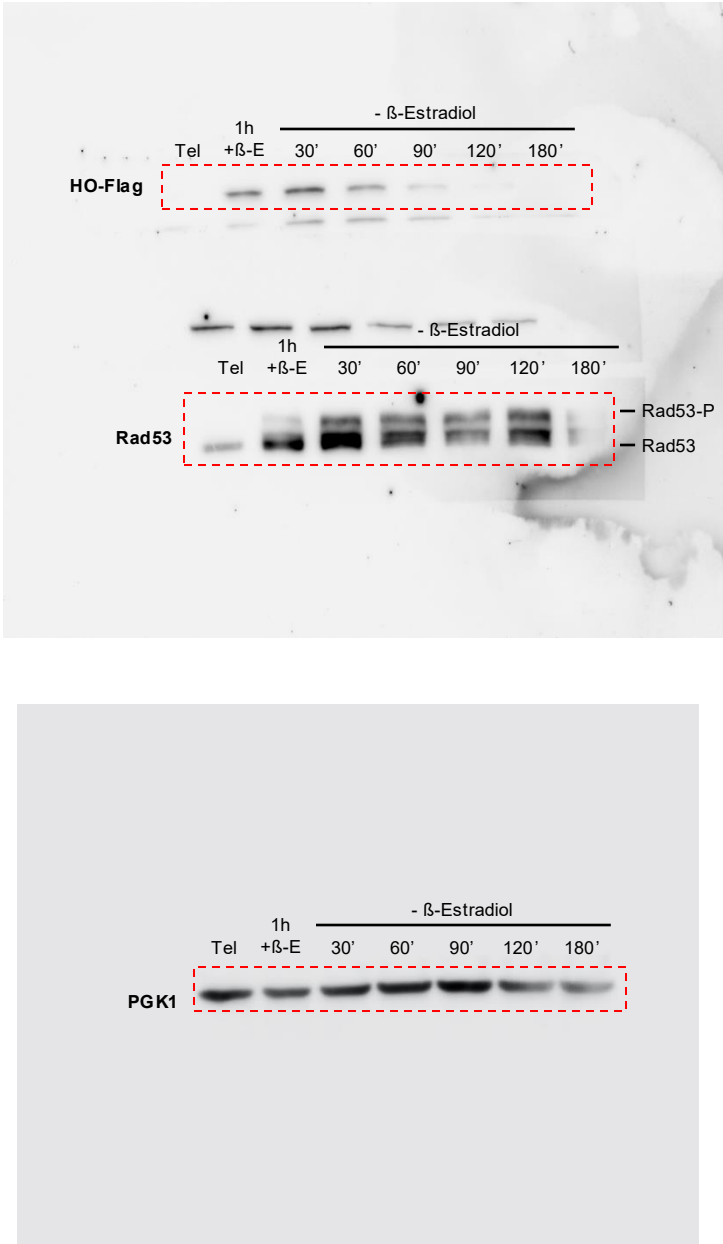

Figure 3e – Southern blot

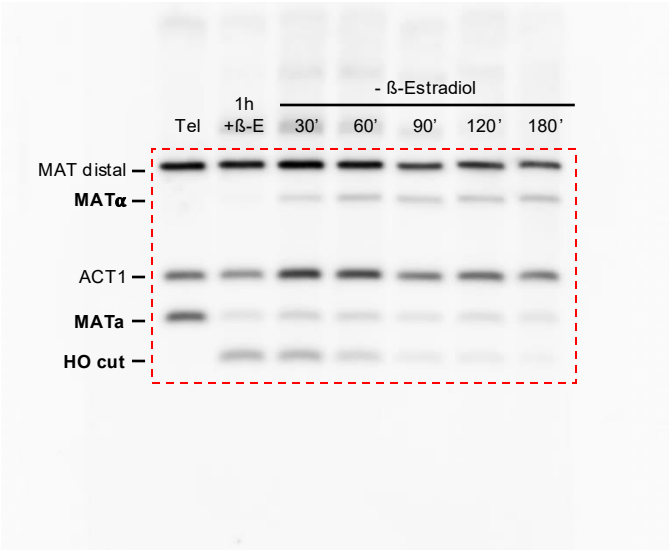

Supplement: Figure 3—source data 2. [file elife-92706-fig3-data2.pdf]

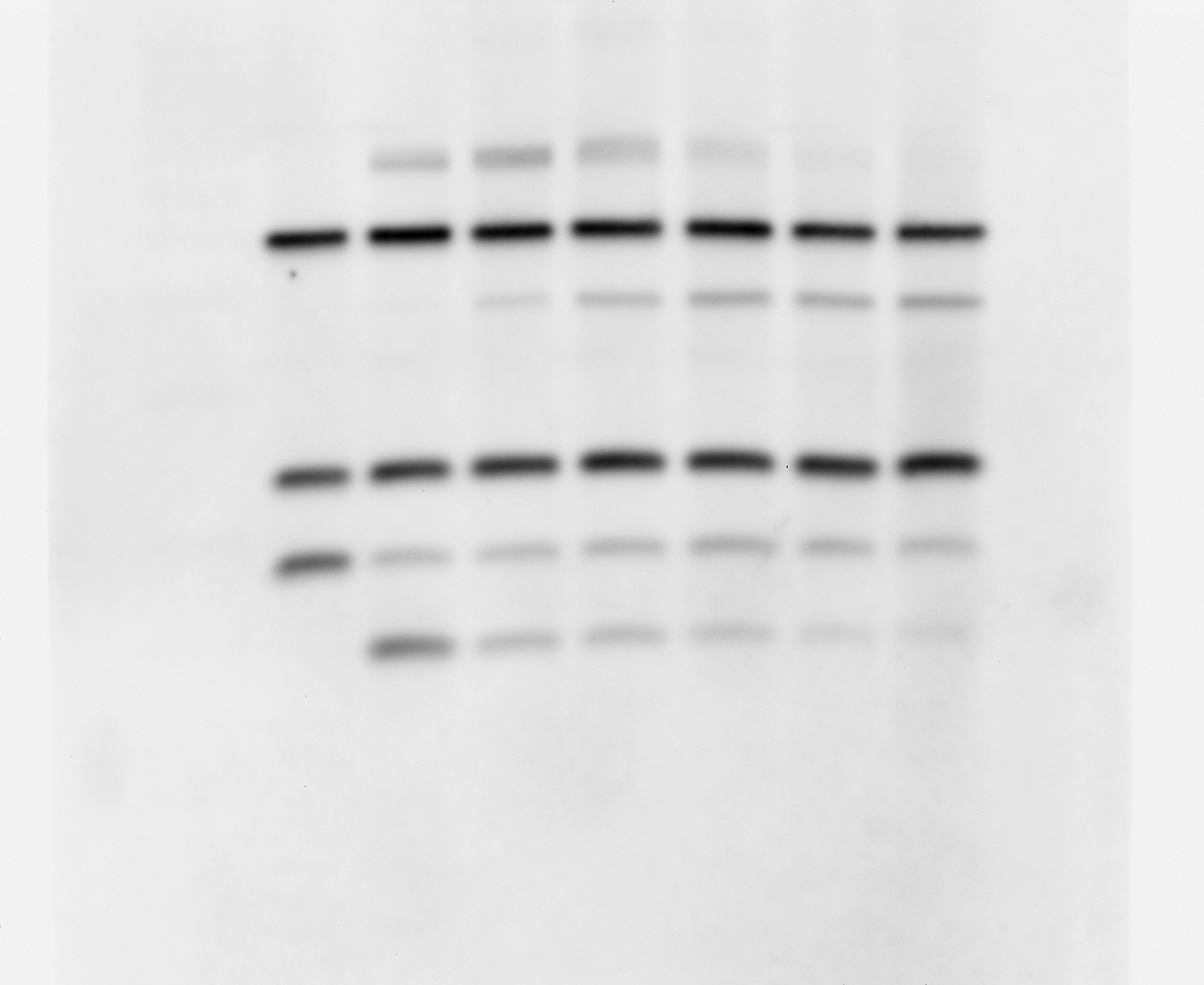

Supplement: Figure 3—figure supplement 2—source data 1. [file elife-92706-fig3-figsupp2-data1.zip › Figure 3 - figure supplement 2 - source data 1_original blots/Fig 3-SF2A_Southern blot Dyku70.TIF]

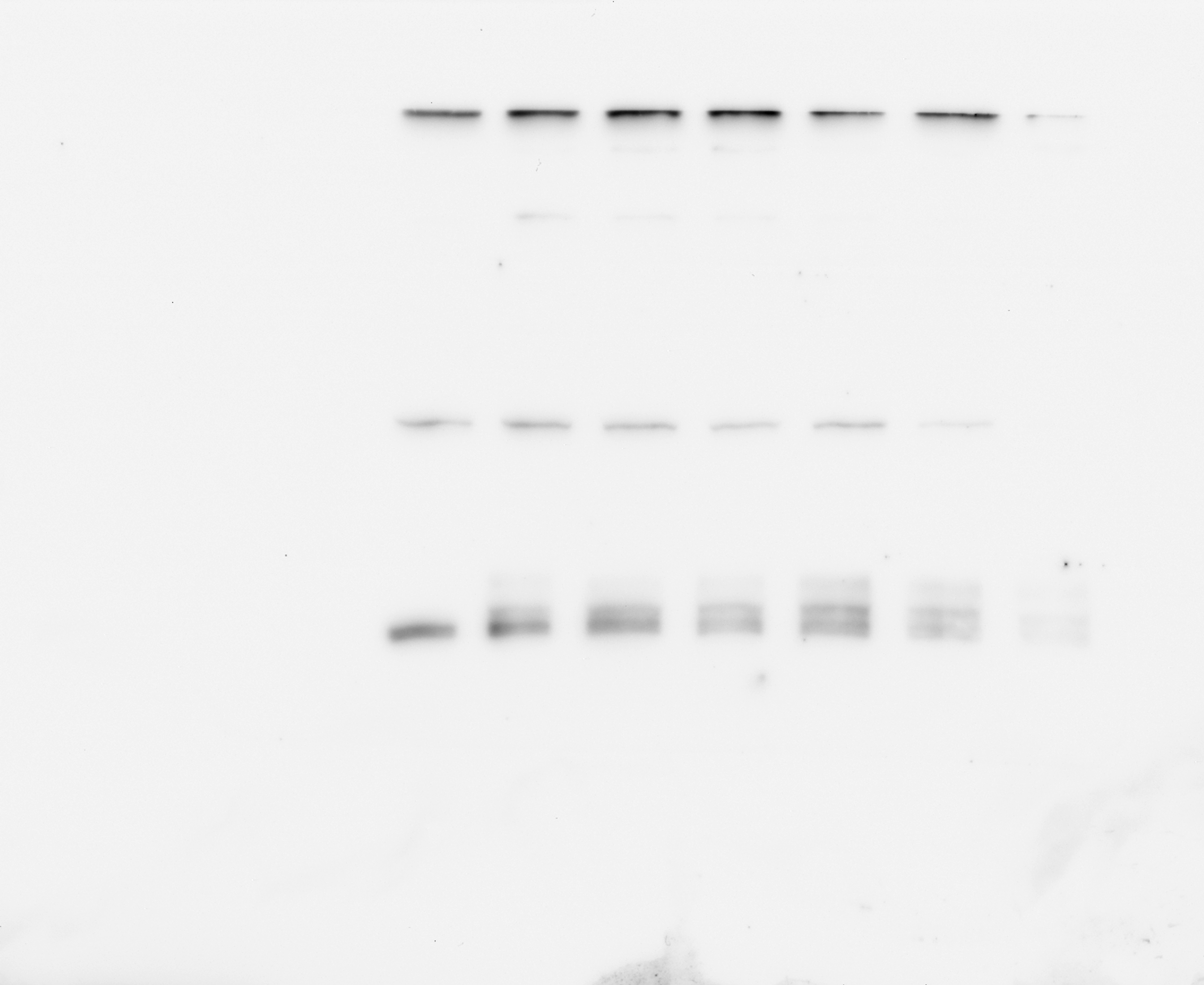

Supplement: Figure 3—figure supplement 2—source data 1. [file elife-92706-fig3-figsupp2-data1.zip › Figure 3 - figure supplement 2 - source data 1_original blots/Fig 3-SF2B. HO-Flag and Rad53.TIF]

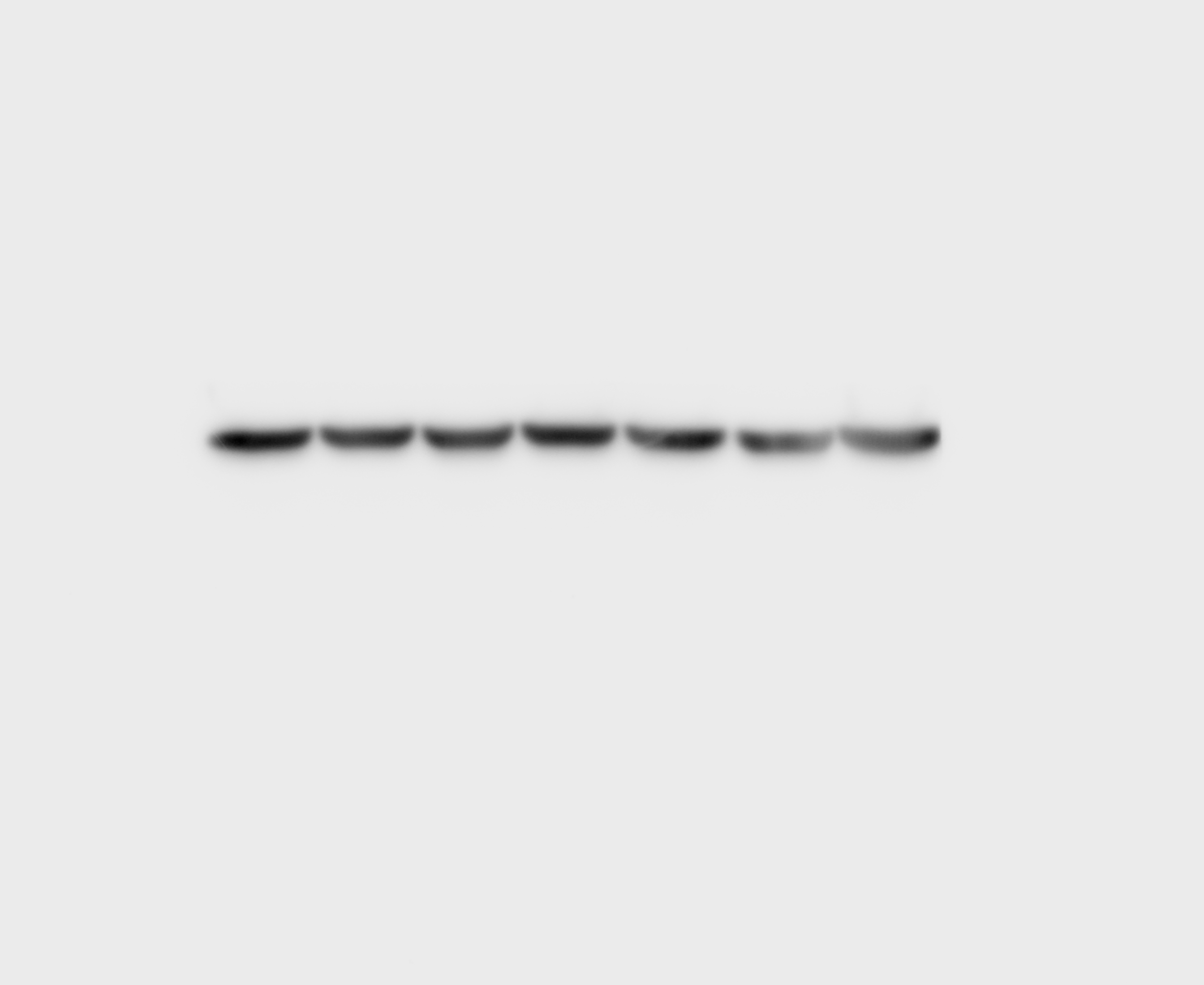

Supplement: Figure 3—figure supplement 2—source data 1. [file elife-92706-fig3-figsupp2-data1.zip › Figure 3 - figure supplement 2 - source data 1_original blots/Fig 3-SF2B. PGK1.tif]

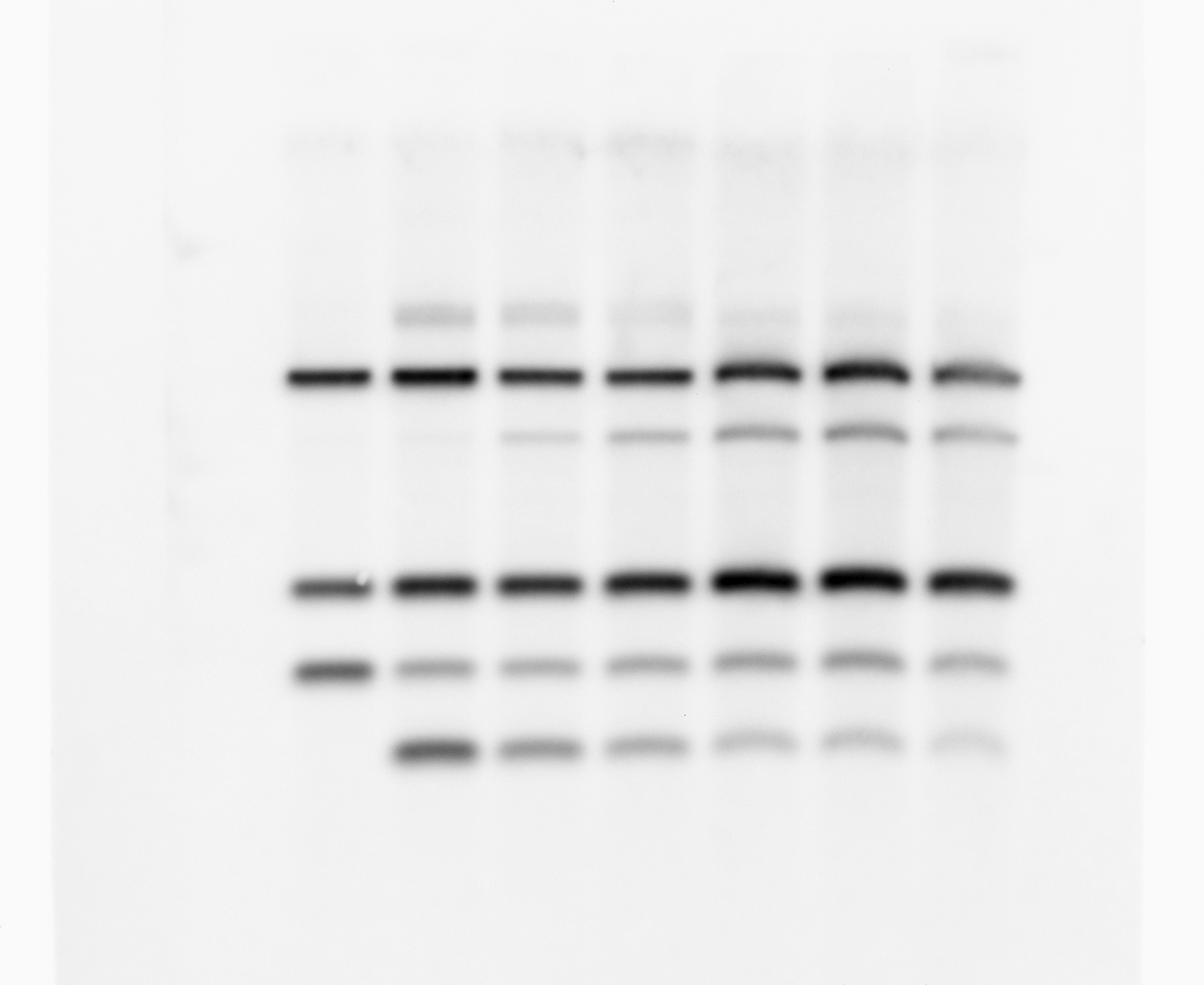

Supplement: Figure 3—figure supplement 2—source data 1. [file elife-92706-fig3-figsupp2-data1.zip › Figure 3 - figure supplement 2 - source data 1_original blots/Fig 3-SF2D. Southern blot Drad9.TIF]

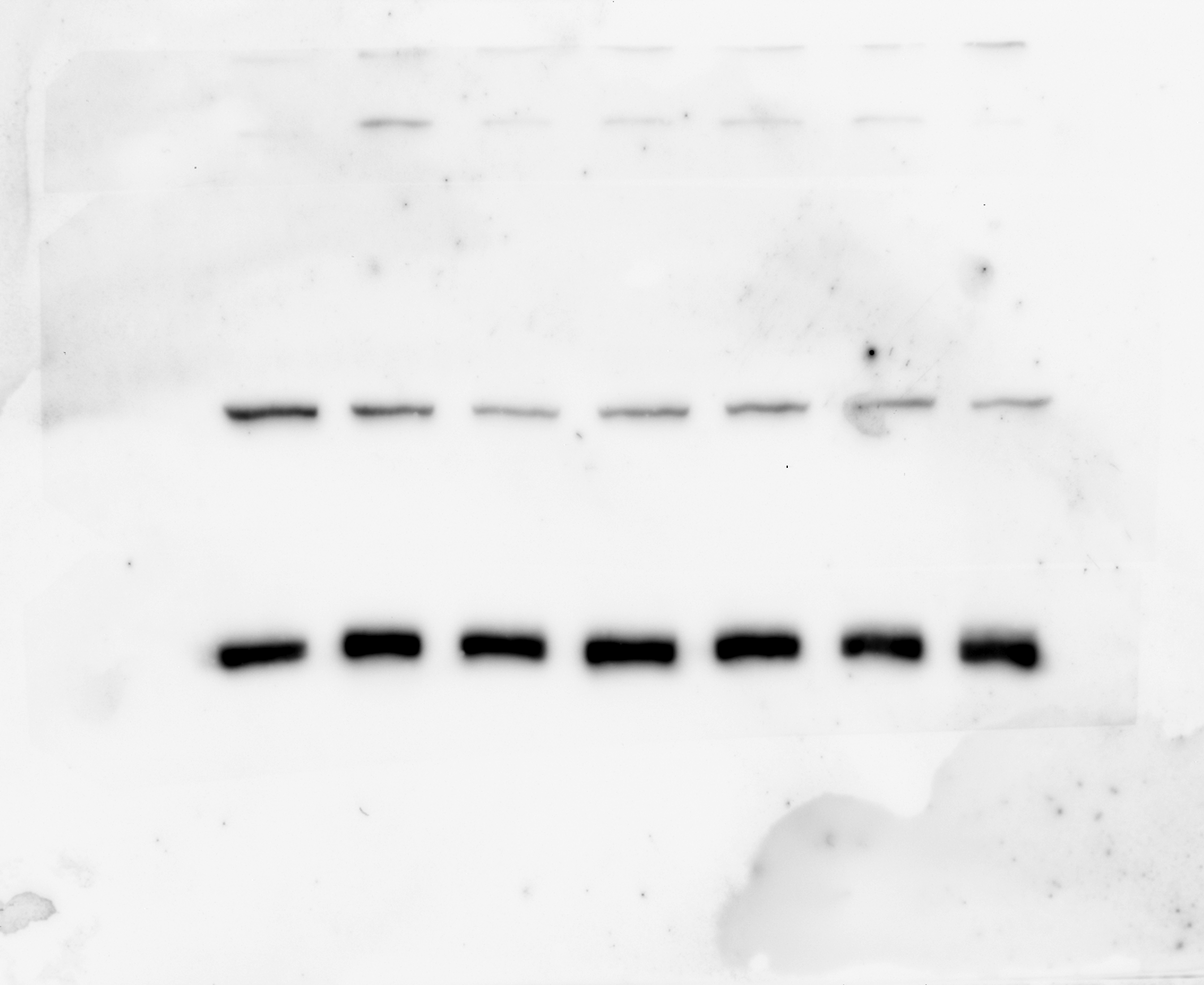

Supplement: Figure 3—figure supplement 2—source data 1. [file elife-92706-fig3-figsupp2-data1.zip › Figure 3 - figure supplement 2 - source data 1_original blots/Fig 3-SF2E. HO-Flag and Rad53.TIF]

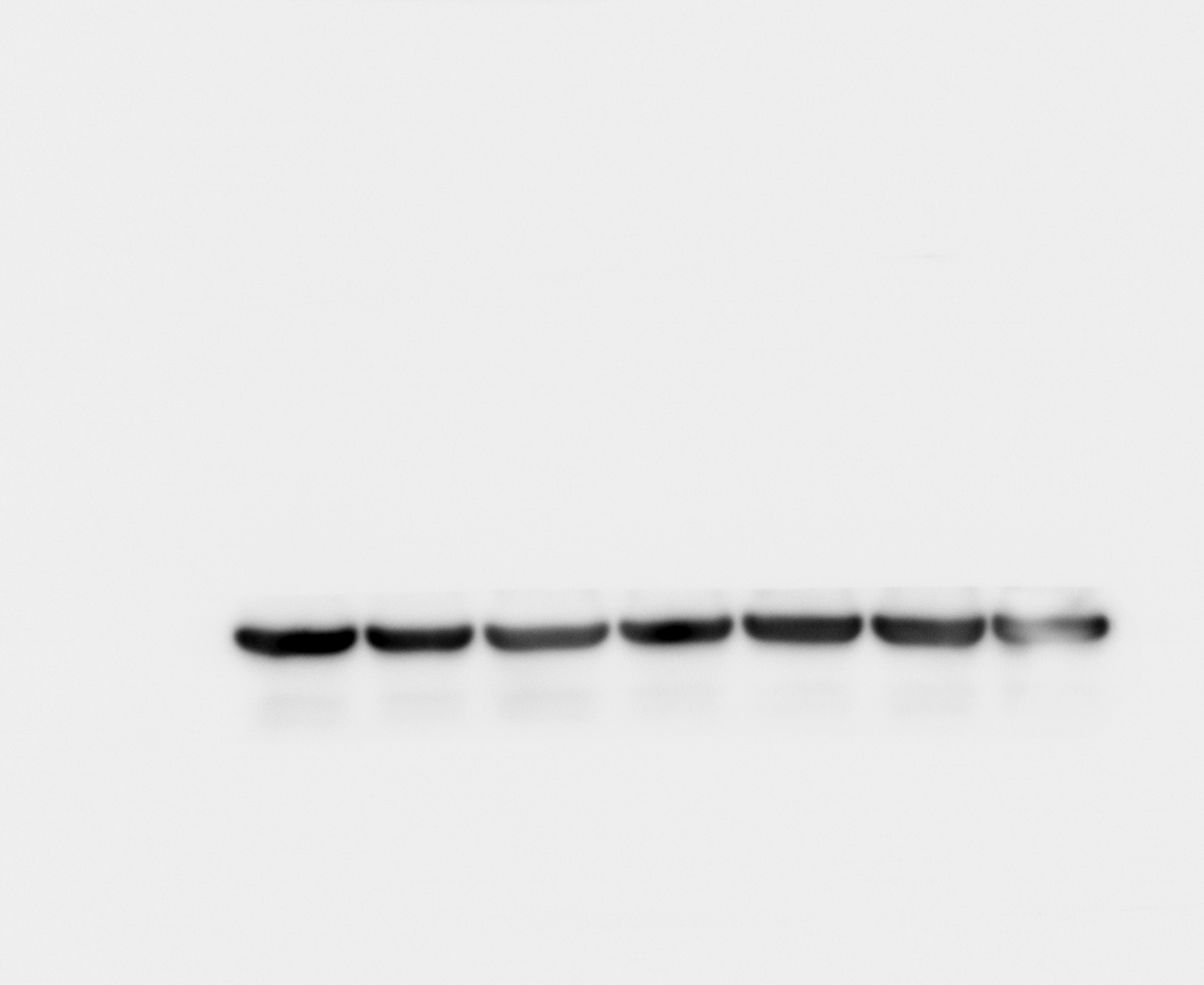

Supplement: Figure 3—figure supplement 2—source data 1. [file elife-92706-fig3-figsupp2-data1.zip › Figure 3 - figure supplement 2 - source data 1_original blots/Fig 3-SF2E. PGK1.TIF]

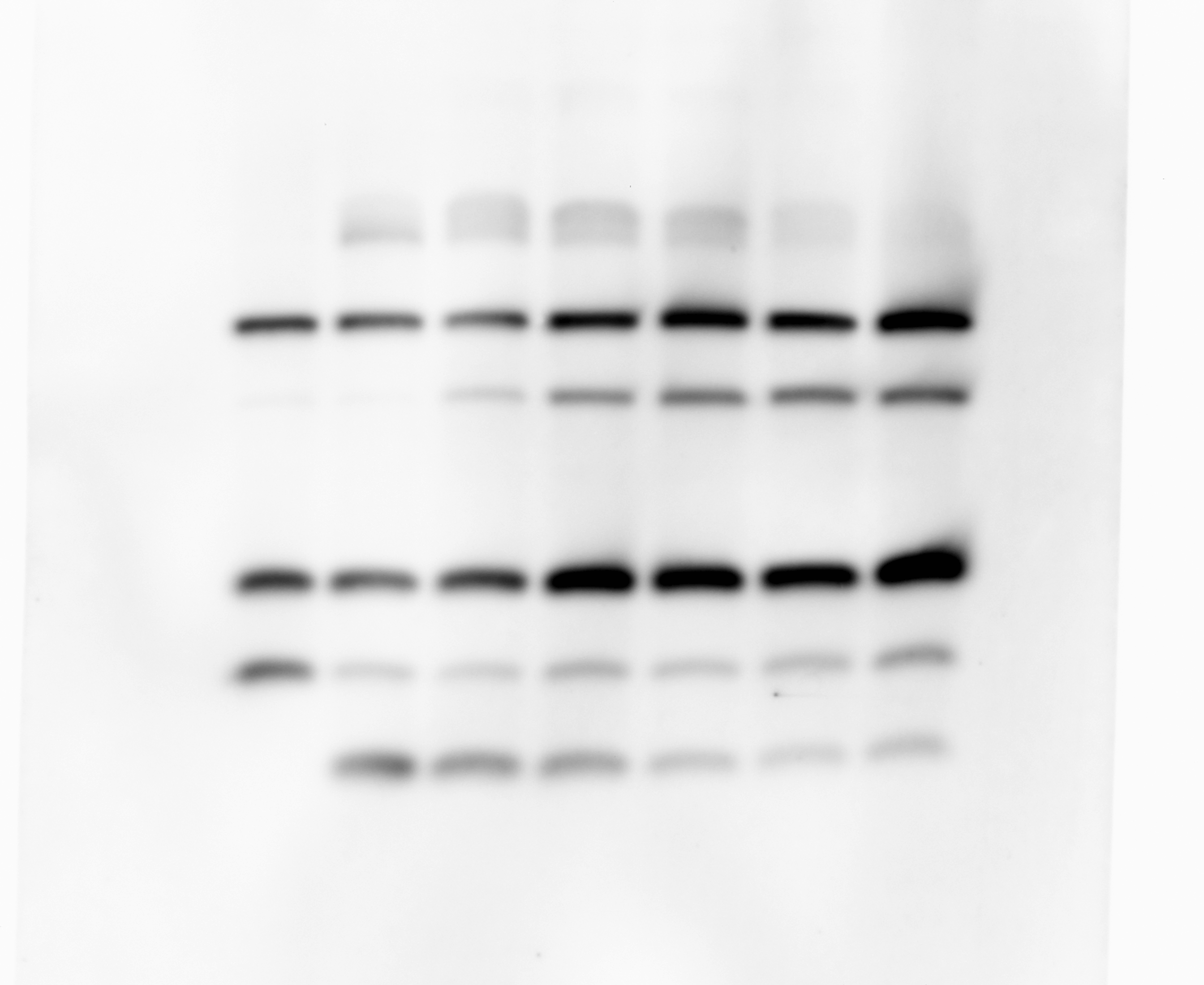

Supplement: Figure 3—figure supplement 2—source data 1. [file elife-92706-fig3-figsupp2-data1.zip › Figure 3 - figure supplement 2 - source data 1_original blots/Fig 3-SF2G. Southern blot Γêåmre11.tiff]

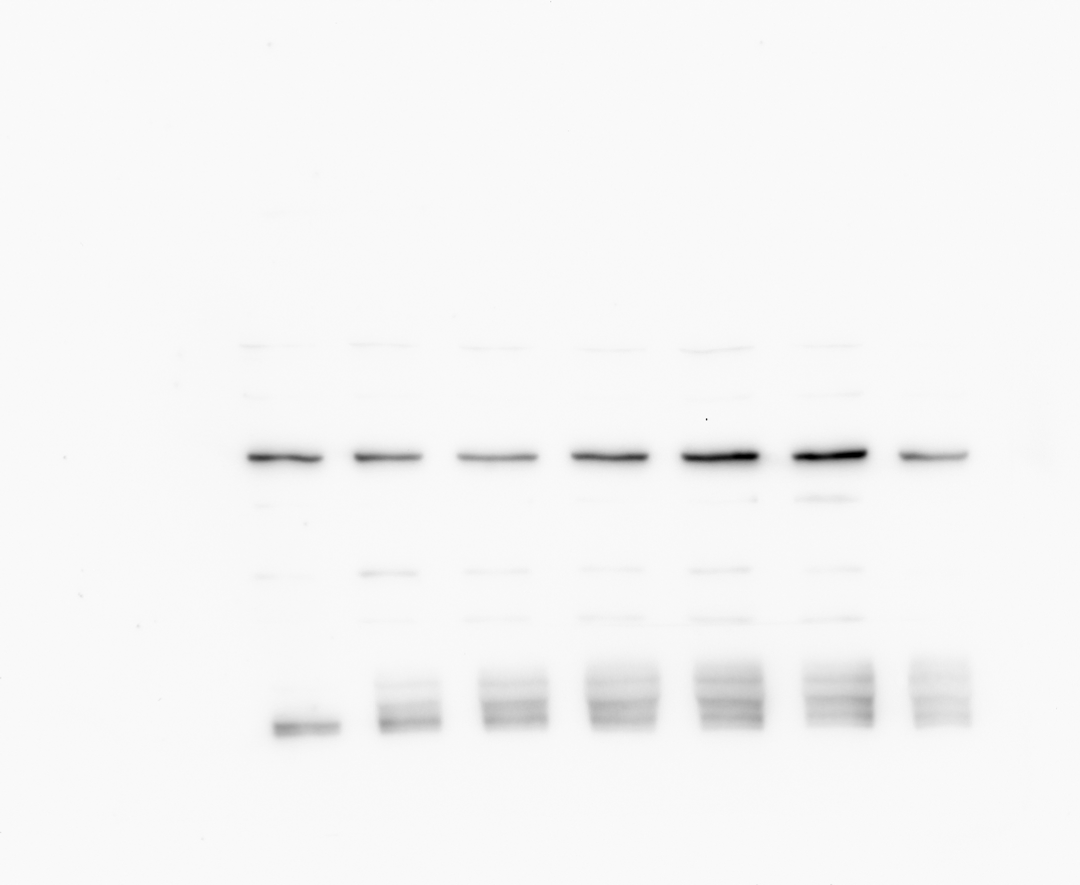

Supplement: Figure 3—figure supplement 2—source data 1. [file elife-92706-fig3-figsupp2-data1.zip › Figure 3 - figure supplement 2 - source data 1_original blots/Fig 3-SF2H. HO-Flag and Rad53.tiff]

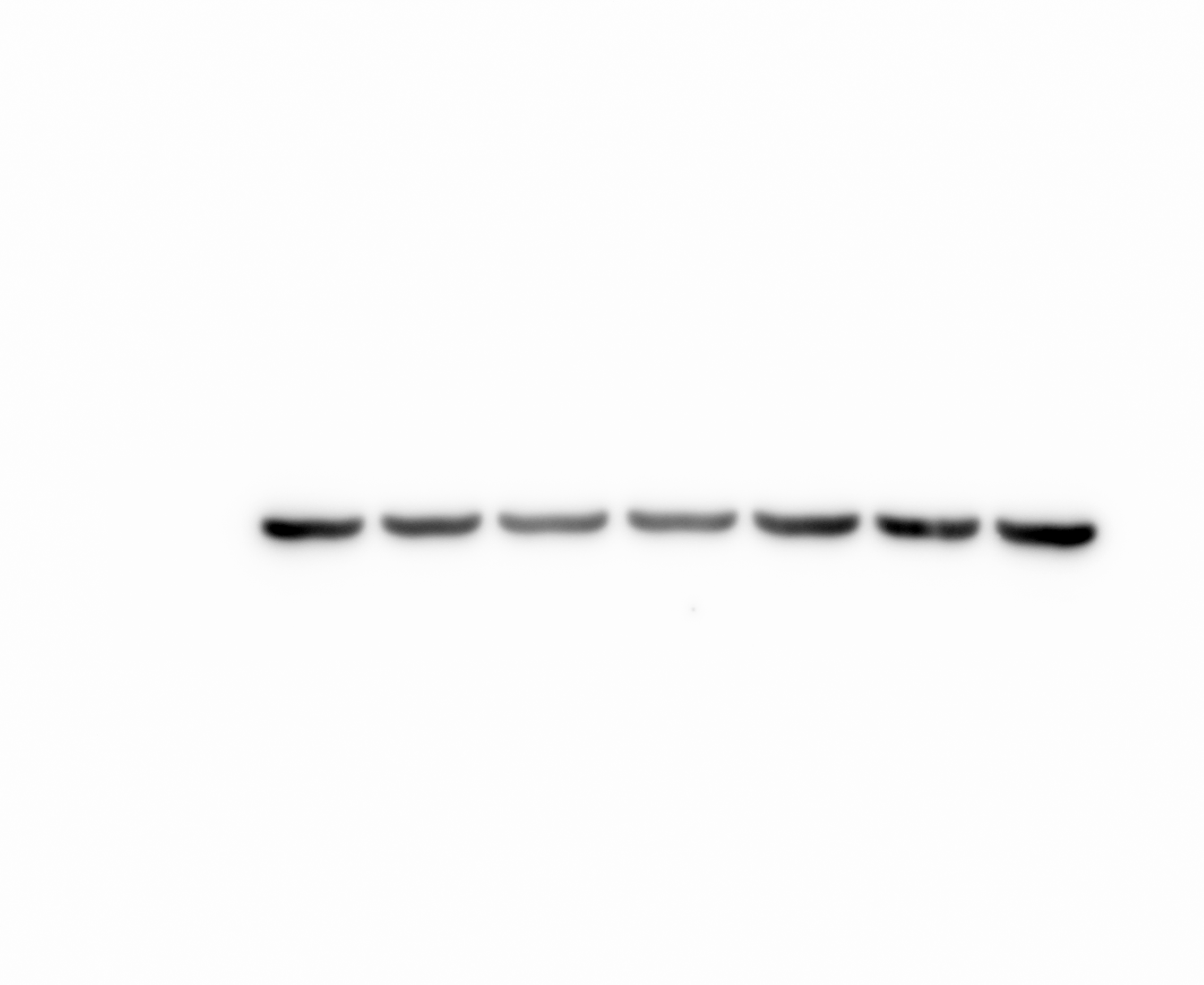

Supplement: Figure 3—figure supplement 2—source data 1. [file elife-92706-fig3-figsupp2-data1.zip › Figure 3 - figure supplement 2 - source data 1_original blots/Fig 3-SF2H. PGK1.tif]

Fig 4B

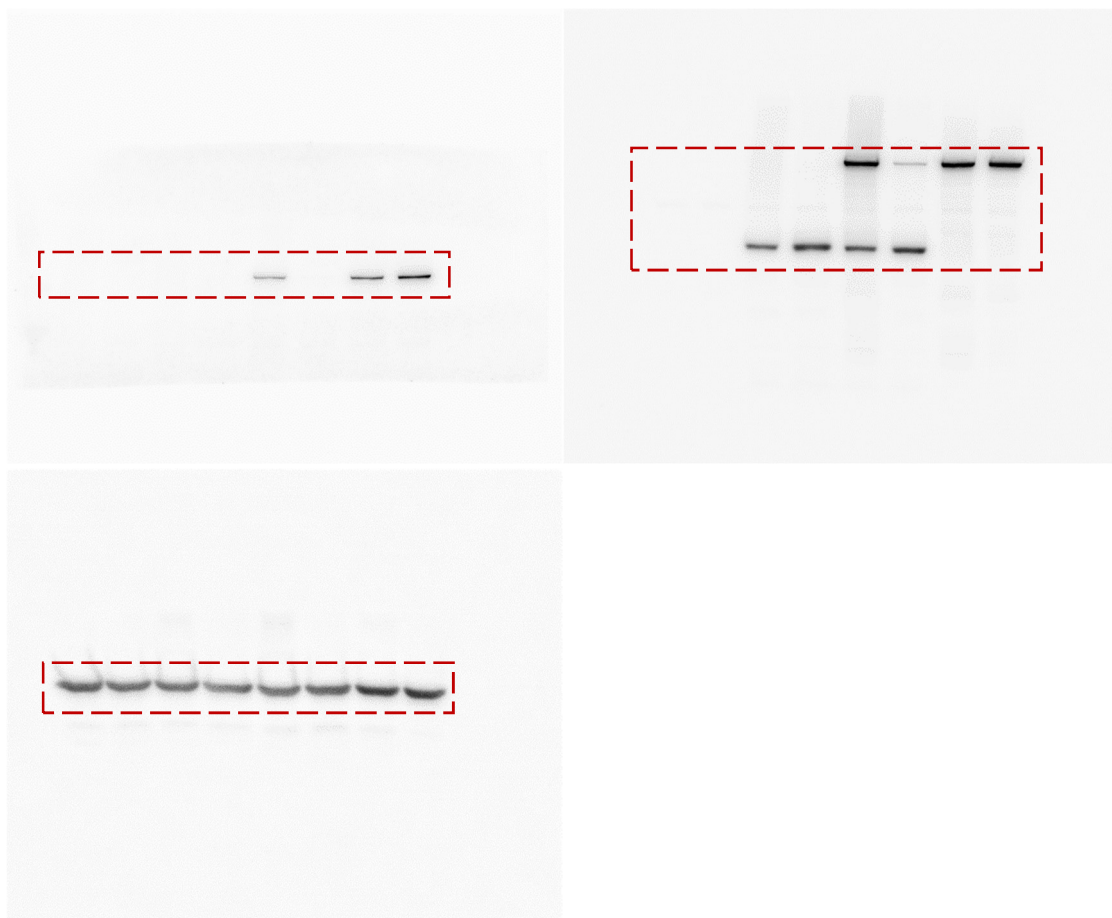

Fig 4C

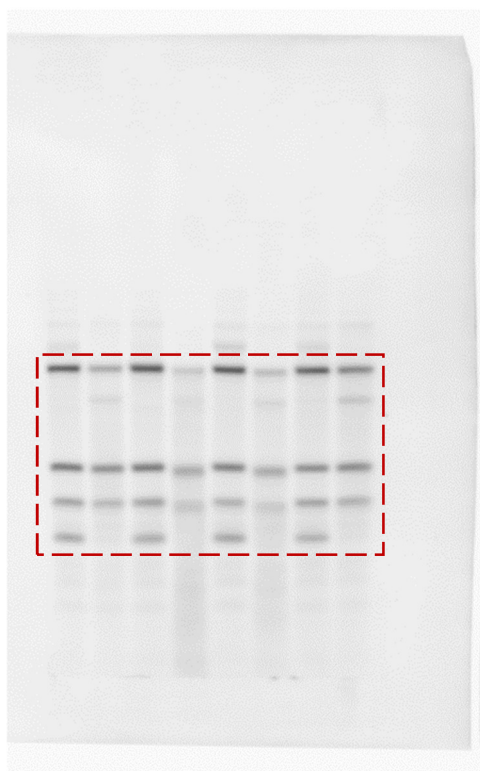

Supplement: Figure 4—source data 2. [file elife-92706-fig4-data2.pdf]

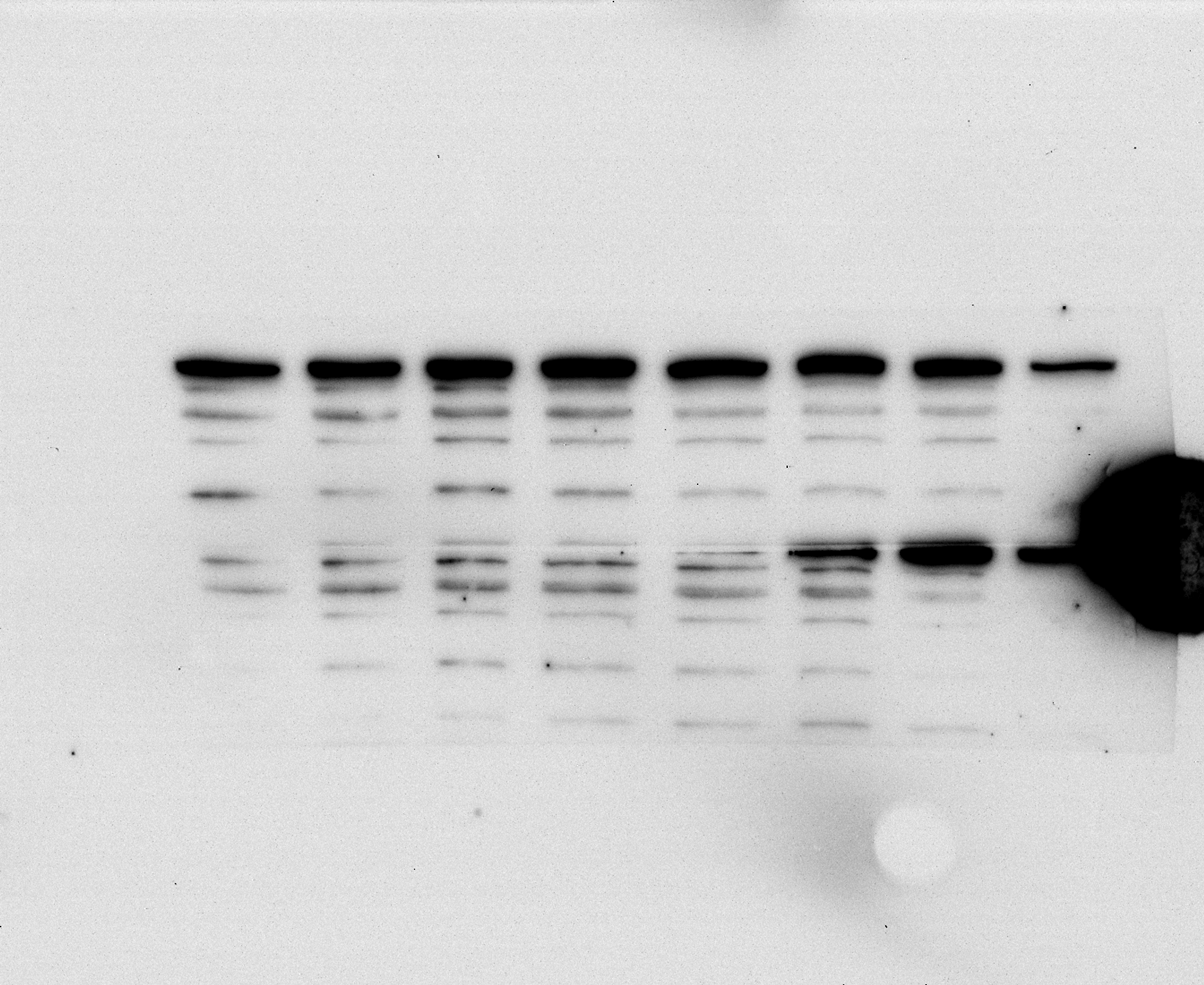

Supplement: Figure 4—figure supplement 1—source data 1. [file elife-92706-fig4-figsupp1-data1.zip › Figure 4 - figure supplement 1 - source data 1_original blots/Fig 4-FS1A. HO-Flag.Tif]

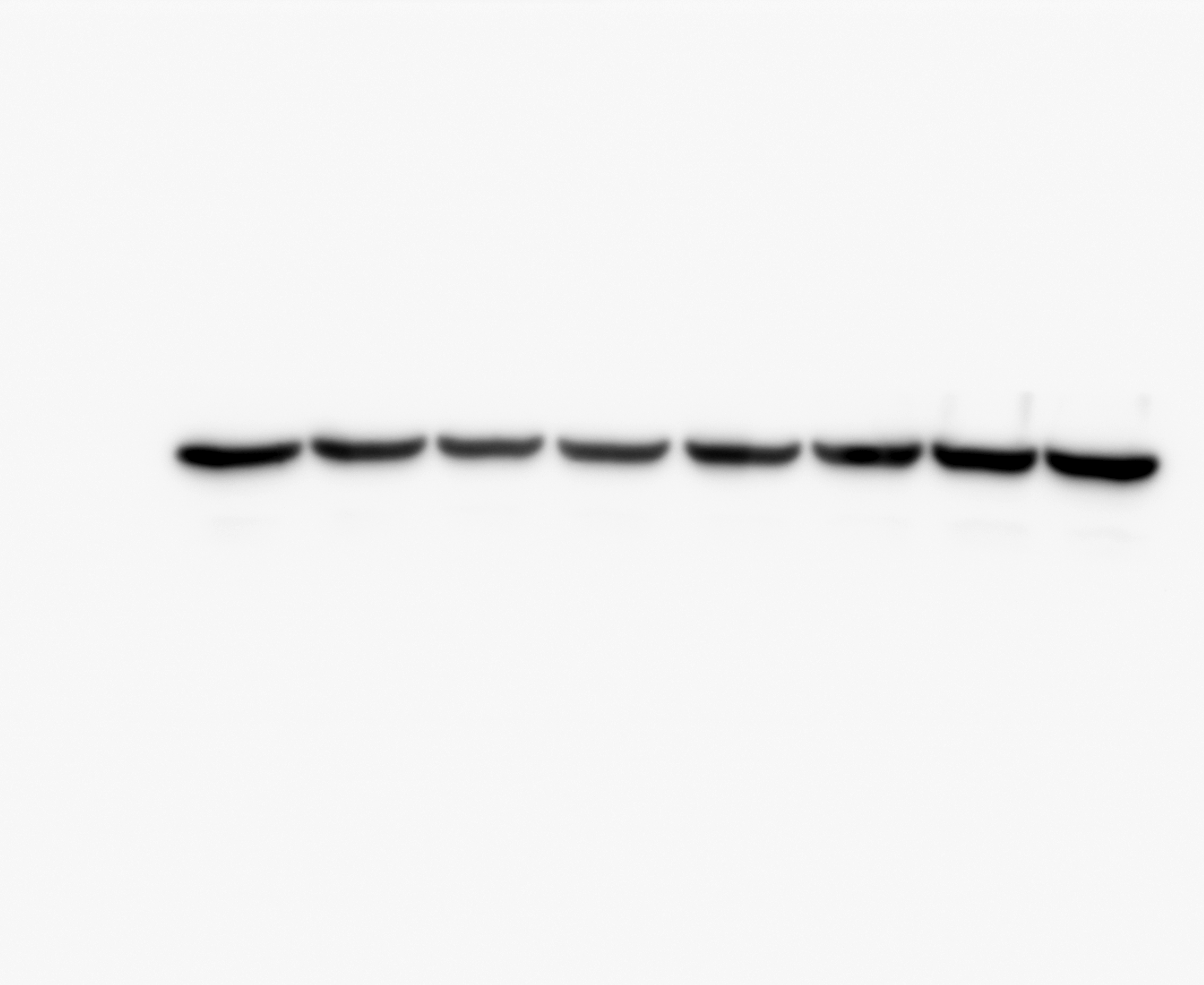

Supplement: Figure 4—figure supplement 1—source data 1. [file elife-92706-fig4-figsupp1-data1.zip › Figure 4 - figure supplement 1 - source data 1_original blots/Fig 4-FS1A. PGK1.TIF]

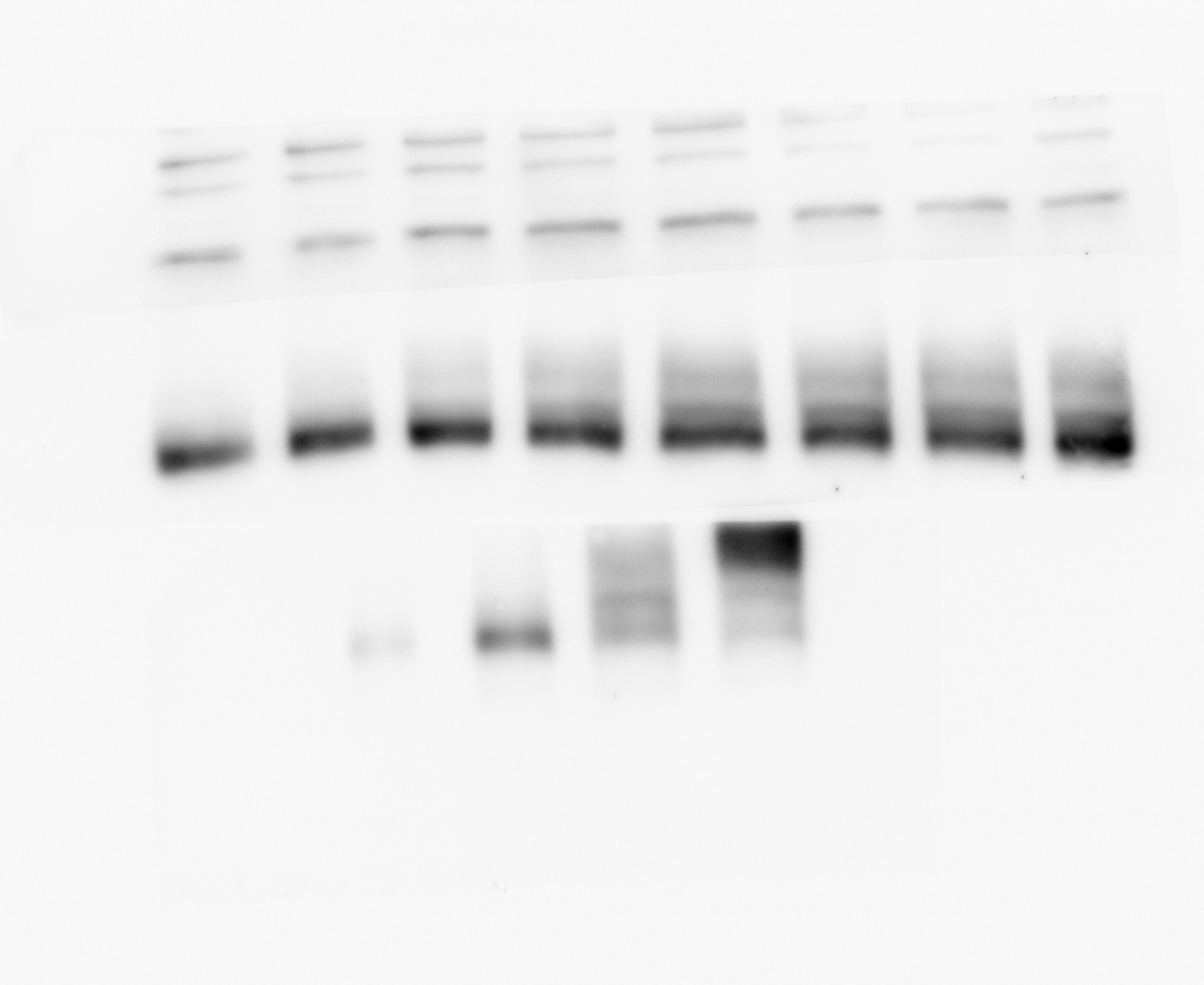

Supplement: Figure 4—figure supplement 1—source data 1. [file elife-92706-fig4-figsupp1-data1.zip › Figure 4 - figure supplement 1 - source data 1_original blots/Fig 4-FS1A. Rad53.tiff]

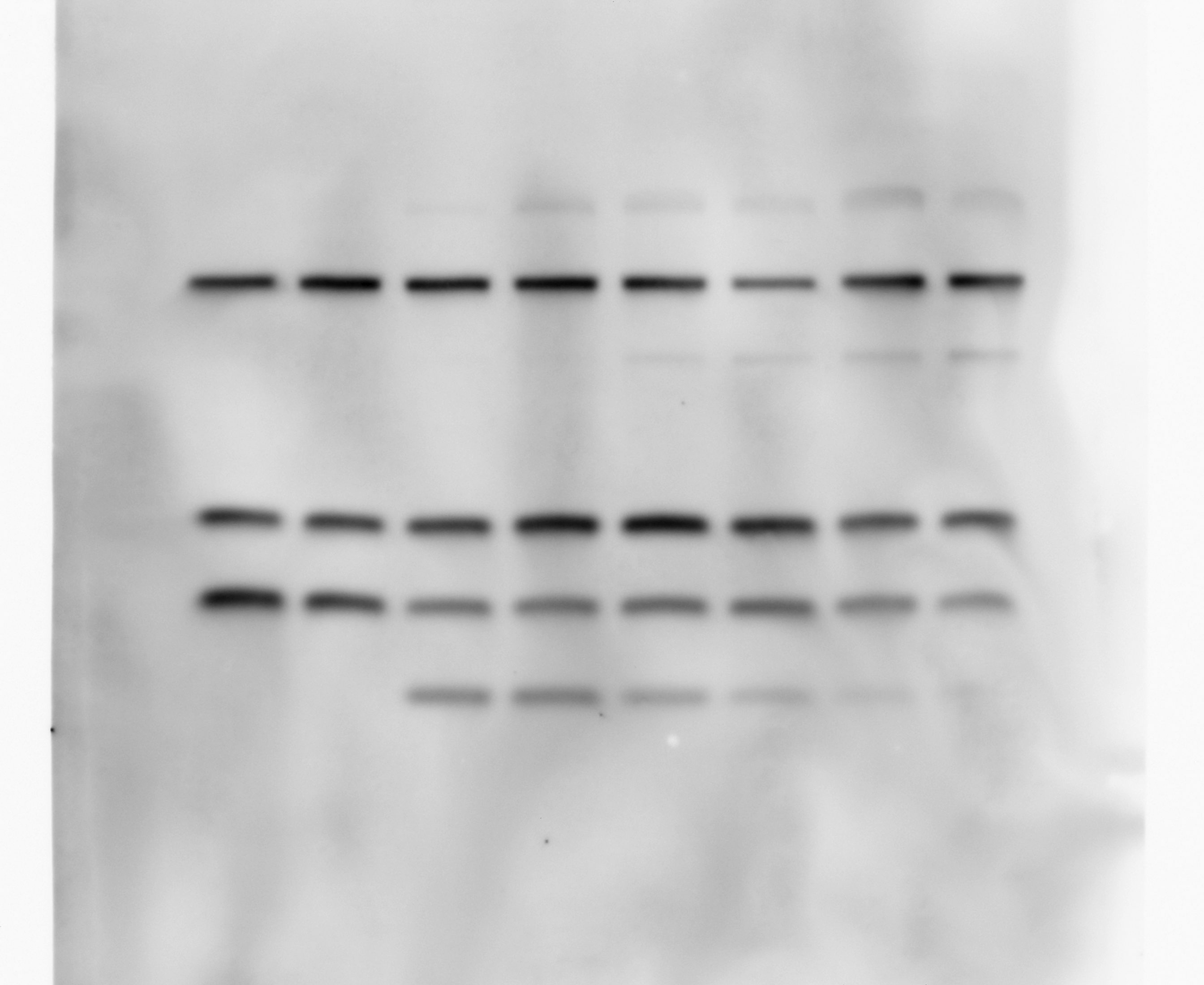

Supplement: Figure 4—figure supplement 1—source data 1. [file elife-92706-fig4-figsupp1-data1.zip › Figure 4 - figure supplement 1 - source data 1_original blots/Fig 4-FS1B. Southern blot.TIF]

Figure S6a – Western blots

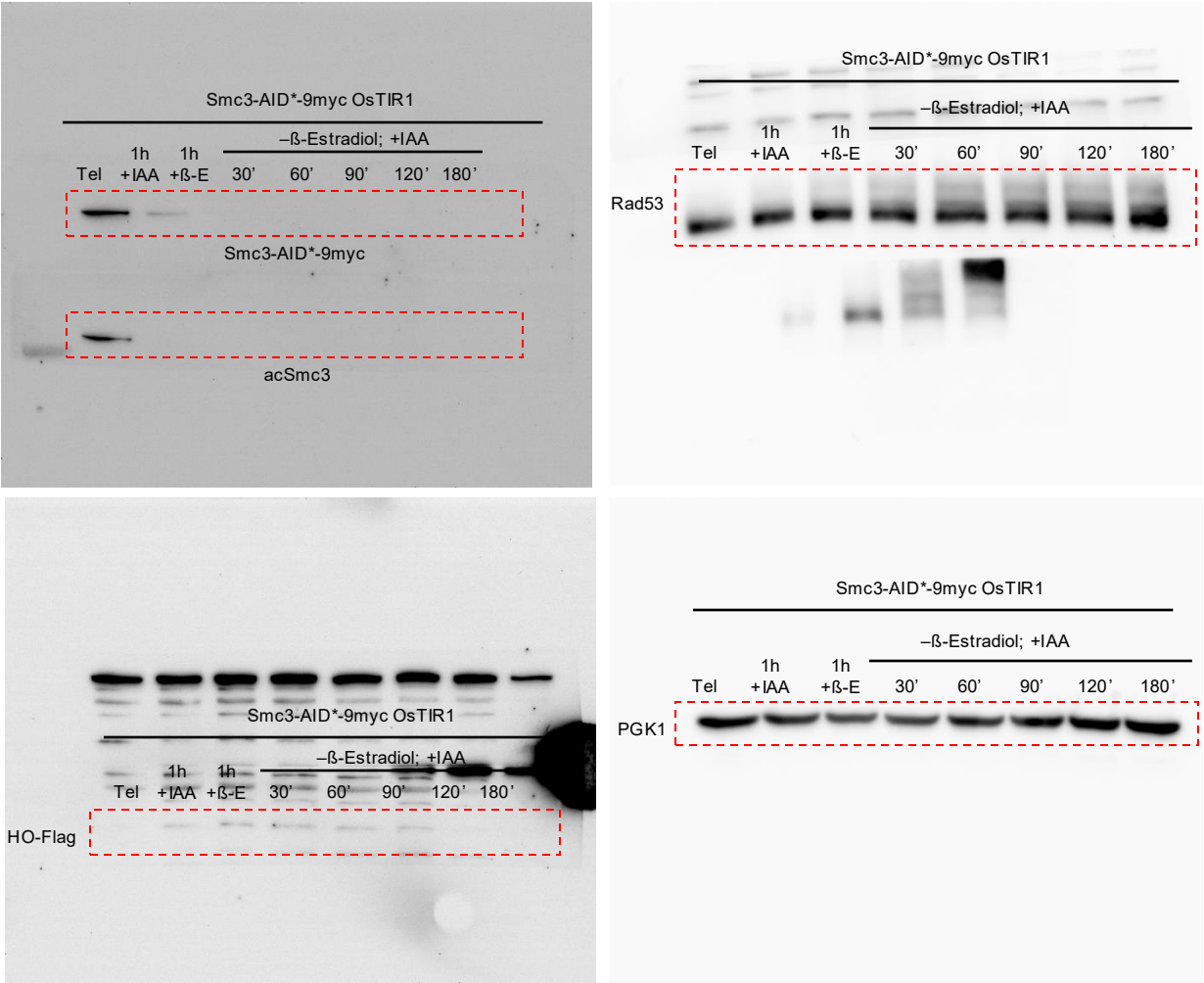

Figure S6b – Southern blot

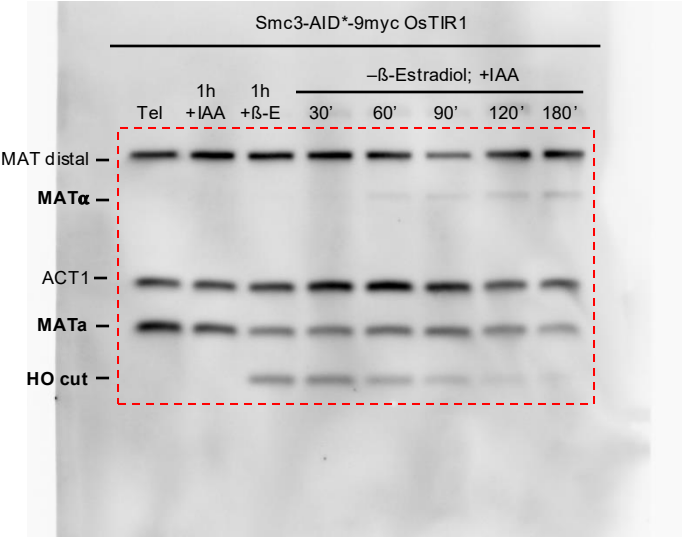

Supplement: Figure 4—figure supplement 1—source data 2. [file elife-92706-fig4-figsupp1-data2.pdf]
