# Supplementary material for: Cohesin reconstitution and homologous recombination repair of DNA double-strand breaks in late mitosis
Source: eLife. 2025 Nov 11;13:RP92706. doi: 10.7554/eLife.92706 (PMC12604858; doi:10.7554/eLife.92706)

Figure S5a – Southern blot

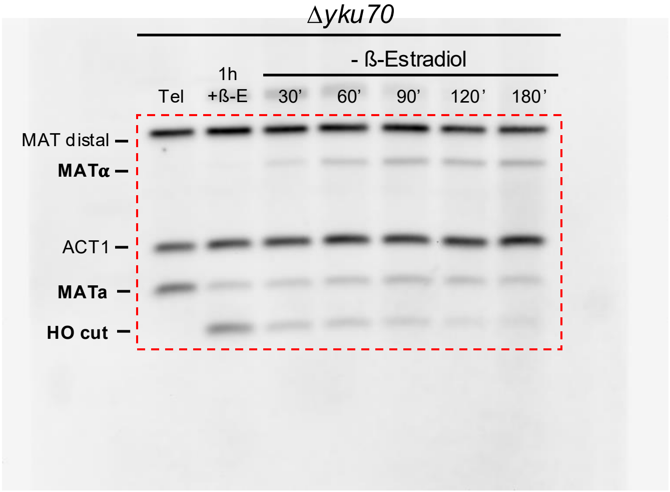

Figure S5b – Western blots

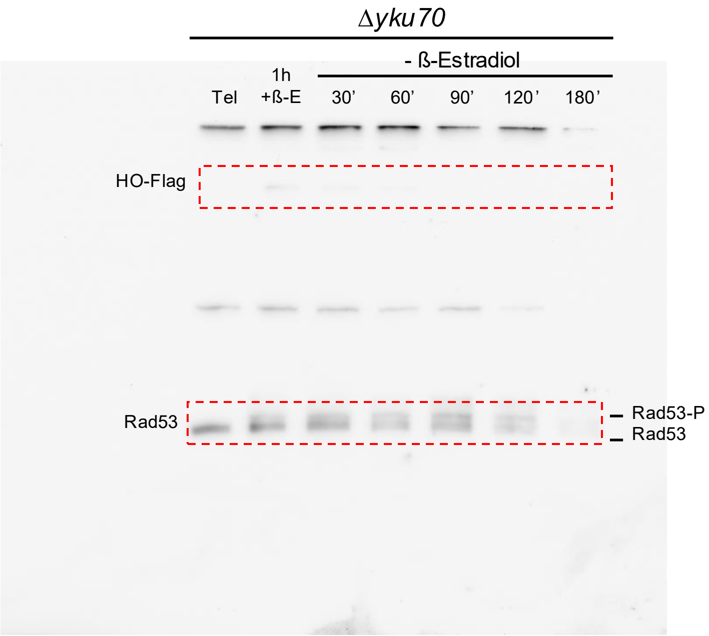

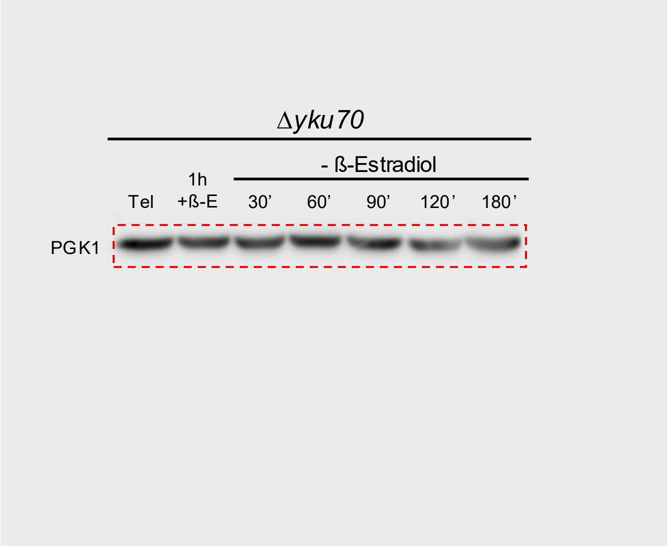

Figure S5d – Southern blot

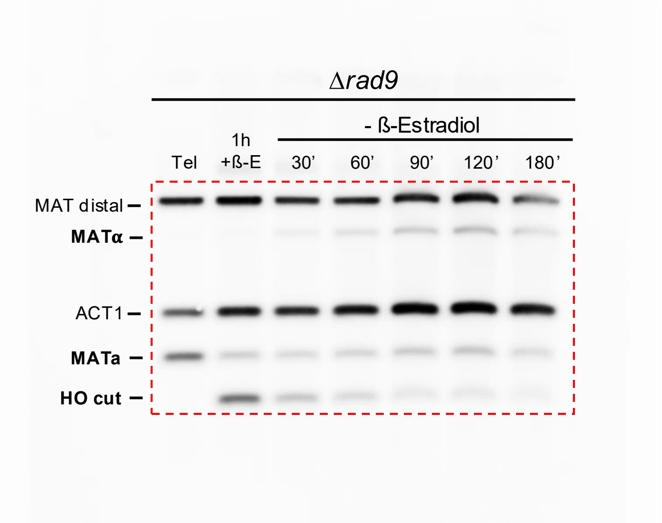

Figure S5e – Western blots

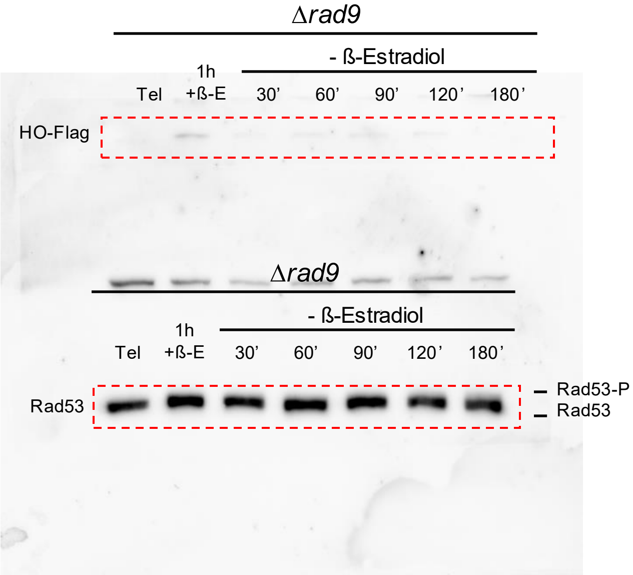

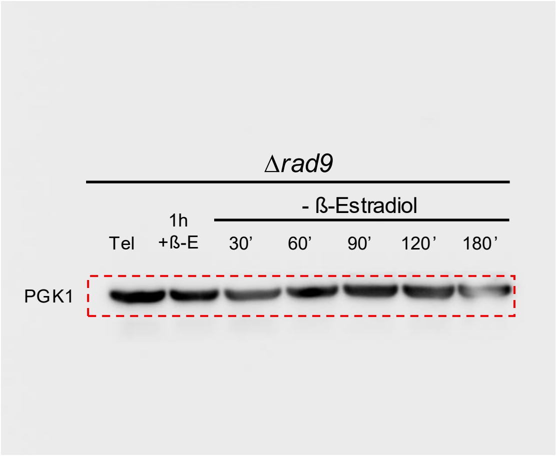

Figure S5g – Southern blot

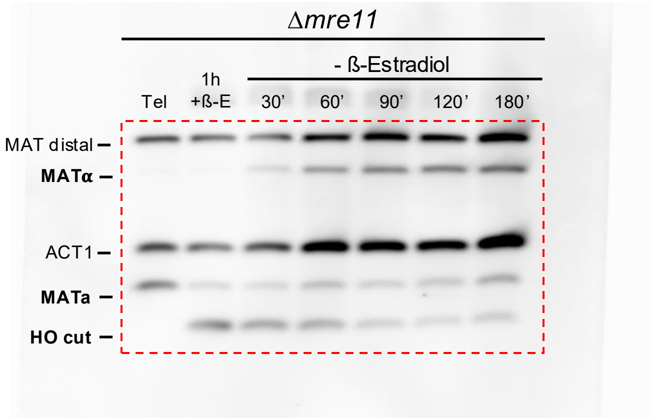

Figure S5h – Western blots

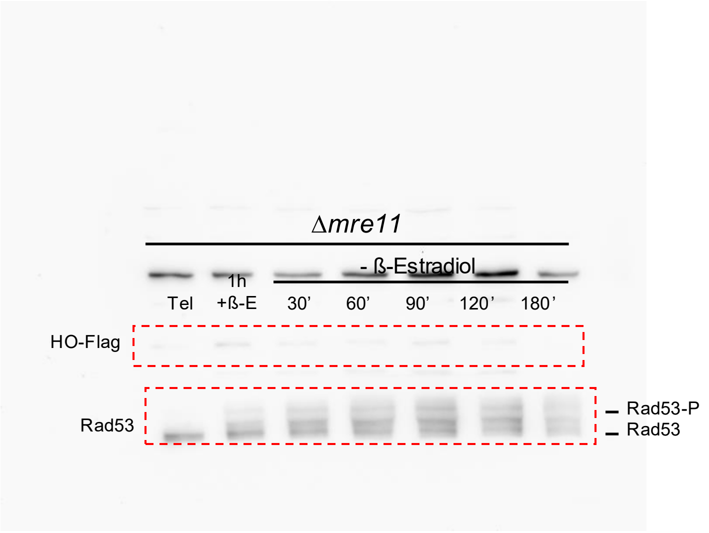

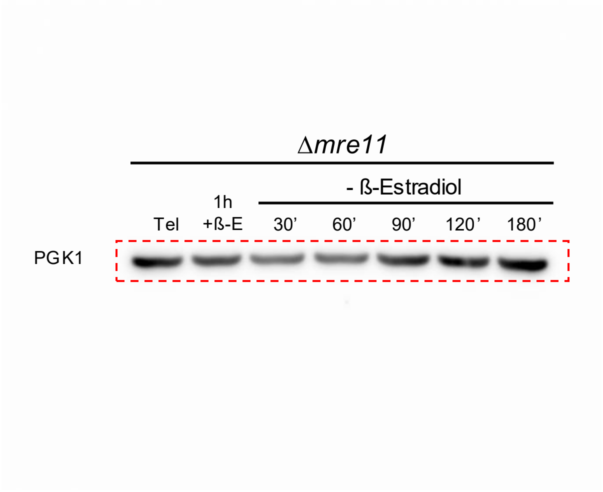

Supplement: Figure 3—figure supplement 2—source data 2. [file elife-92706-fig3-figsupp2-data2.pdf]
